# Estimation of the in vivo neutralization potency of eCD4Ig and conditions for AAV-mediated production for SHIV long-term remission

**DOI:** 10.1126/sciadv.abj5666

**Published:** 2022-01-12

**Authors:** Ashish Goyal, Matthew Gardner, Bryan T. Mayer, Keith R. Jerome, Michael Farzan, Joshua T. Schiffer, E. Fabian Cardozo-Ojeda

**Affiliations:** 1 Vaccine and Infectious Diseases Division, Fred Hutchinson Cancer Research Center, Seattle, WA, USA.; 2 Department of Medicine, Emory University, Atlanta, GA, USA.; 3 Department of Laboratory Medicine and Pathology, University of Washington, Seattle, WA, USA.; 4 Department of Immunology and Microbiology, Scripps Research Institute, Florida Campus, Jupiter, FL, USA.; 5 Department of Medicine, University of Washington, Seattle, WA, USA.; 6 Clinical Research Division, Fred Hutchinson Cancer Research Center, Seattle, WA, USA.

## Abstract

The engineered protein eCD4Ig has emerged as a promising approach to achieve HIV remission in the absence of antiviral therapy. eCD4Ig neutralizes nearly all HIV-1 isolates and induces antibody-dependent cell-mediated cytotoxicity (ADCC) in vitro. To characterize the in vivo antiviral neutralization and possible ADCC effects of eCD4Ig, we fit mathematical models to eCD4Ig, anti–eCD4Ig-drug antibody (ADA), and viral load kinetics from healthy and simian-human immunodeficiency virus AD8 (SHIV-AD8) infected nonhuman primates that were treated with single or sequentially dosed eCD4Ig passive administrations. Our model predicts that eCD4Ig transiently decreases SHIV viral loads due to neutralization only with an in vivo IC_50_ of ~25 μg/ml but with limited effect due to ADA. Simulations suggest that endogenous, continuous expression of eCD4Ig at levels greater than 10^5^ μg/day, as is possible with Adeno-associated virus (AAV) vector-based production, could overcome the diminishing effects of ADA and allow for long-term remission of SHIV viremia in nonhuman primates.

## Teaser

eCD4Ig has an in vivo IC_50_ of 25 μg/ml, and it may induce ART-free virus control if produced continually at 10^5^ μg/day or more.

## INTRODUCTION

Antiretroviral therapy (ART) suppresses HIV viremia and extends human lives for decades and has long served as the backbone for HIV therapy. However, ART must be taken daily, which has prompted a search for different avenues to achieve ART-free remission including those that incorporate broadly neutralizing antibodies (bNAbs) (*1*–*4*). bNAbs not only prevent cell-free virus from entering cells but also can enhance the clearance of infected cells via antibody-dependent cell-mediated cytotoxicity (ADCC) in addition to possibly preserving helper CD4^+^ T cells, stimulating effective CD8^+^ T cell response and preventing the emergence of antibody-resistant virus (*5*, *6*). Working on a similar principle but with superior breadth relative to bNAbs (*7*–*9*), eCD4Ig is a synthetic antibody-like inhibitor designed to limit HIV entry into CD4^+^/CCR5^+^ cells. This inhibitor closely mimics HIV-1’s obligate receptors and is a promising therapeutic approach against a very diverse array of HIV variants (*7*, *10*). eCD4Ig potency arises due to its capacity to avidly bind both the CD4 and coreceptor binding sites of the HIV-1 envelope glycoprotein (Env) trimer (*8*) and its breadth is a function of binding both conserved and functionally important Env regions (*10*). In addition to preventing viral entry, eCD4Ig can also mediate the killing of HIV-infected cells via ADCC and, thus, may help reduce the burden of latently infected cells (*11*). The promising results of eCD4Ig in animal models have accelerated its potential as an agent for HIV prevention and therapy and potentially as part of a functional cure regimen (*7*, *10*).

The observed potent effects of eCD4Ig may potentially be compromised by the emergence of antidrug antibodies (ADAs) resulting from recognition of eCD4Ig by memory B cells as foreign entity (*7*, *12*, *13*). However, the dynamics of interplay between ADA and eCD4Ig are not fully understood, particularly in an in vivo setting. It has also been challenging to estimate the relative importance of eCD4Ig-induced ADCC in vivo. This lack of knowledge could possibly result in suboptimal dosing strategies.

To overcome this challenge, we developed a mathematical model that recapitulates eCD4Ig levels and ADA to analyze pharmacokinetics (PK) dynamics in 21 uninfected rhesus macaques (RMs) and identified the interaction between ADA and eCD4Ig. We then used the same model to reproduce PK dynamics in 12 SHIV-AD8–infected RMs. We next modeled SHIV dynamics and PK dynamics together in the same set of animals. Last, we projected the levels of eCD4Ig needed with a viral vector production approach to continually suppress SHIV viremia.

## RESULTS

### Study design

We analyzed data from two groups of RMs infused with different variants of eCD4Ig that were optimized for thermostability (table S1). The first group comprised 21 healthy RMs administered with four variants of eCD4Ig at three different doses. Two eCD4Ig variants contained the wild-type immunoglobulin G1 (IgG1) and IgG2 Fc sequences. The other two variants contained the M428L/N434S amino acid substitutions in the IgG1 or IgG2 Fc (LS versions) that have been shown to increase the half-life of antibodies (*14*). From this group, we analyzed serum concentration of eCD4Ig and ADA against eCD4Ig. The second group included 12 RMs intravenously challenged with SHIV-AD8 and followed by a single infusion or three infusions of two variants of eCD4Ig at different stages of infection. From the second group, we analyzed serum concentration of eCD4Ig and plasma viral load. We also analyzed serum concentration of eCD4Ig and against eCD4Ig in a third group composed of eight healthy RMs that received eCD4Ig in the form of AAV vectors. We developed mathematical models to (i) characterize the coupling of eCD4Ig PK with the observed ADA response, (ii) characterize the SHIV virus dynamics in the absence and presence of eCD4Ig, (iii) quantify estimates of the in vivo potency of eCD4Ig, and (iv) finally recapitulate eCD4Ig and ADA PK when eCD4Ig is administered in the AAV vector form.

### Kinetics of eCD4Ig and ADA response in healthy RMs

**Fig. 1.**
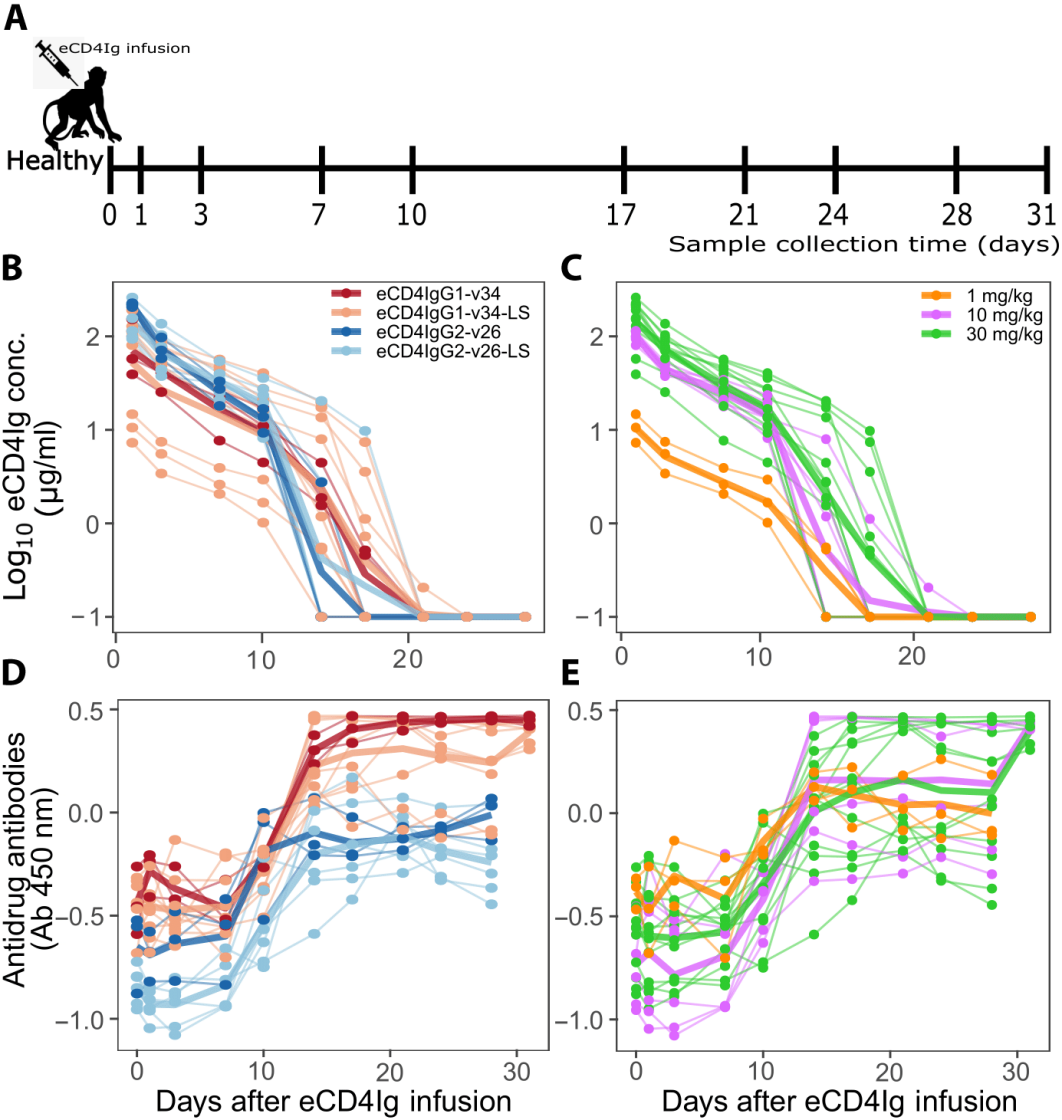
PK of eCD4Ig and ADA after a single infusion in healthy (uninfected) RMs. (**A**) Experimental procedure in healthy RMs. (**B** and **C**) Dynamics of eCD4Ig (log_10_, μg/ml) and (**D** and **E**) ADA (log_10_; Ab, 450 nm) serum concentrations from 21 healthy RMs after a single dose of eCD4Ig at day “0” according to (B and D) eCD4Ig variant and (C and E) infused dose. Solid thick lines represent the mean of the group.

We analyzed the kinetics of eCD4Ig in the presence of ADA from 21 healthy RMs after administering four variants of eCD4Ig at three different doses. Nine animals in groups of three received eCD4IgG1-v34-LS in three different doses at 30, 10, and 1 mg/kg; three animals received eCD4IgG1-v34 at 30 mg/kg; six animals in groups of three received eCD4IgG2-v26-LS at 30 and 10 mg/kg; and three animals received eCD4IgG2-v26 at 30 mg/kg (Fig. 1). Versions v34 for eCD4IgG1 and v26 for eCD4IgG2 were optimized versions of the wild-type Fc sequences for thermostability and manufacturability. In all cases, the serum levels of eCD4Ig had an initial biphasic decline of approximately 1 to 2 logs for the first 10 days after infusion, followed by a fast decline during the following 5 to 10 days and generally dropped below the detection limit by day 21. The ADA levels had an initial stable phase for the first 5 to 7 days, followed by a rapid expansion concurrent with the third eCD4Ig fast-decline phase of more than one order of magnitude. After the expansion, the ADA levels reach a steady-state level that was ~1 log_10_ higher than the baseline for all variants except for the eCD4IgG2-v26 variant, for which the observed increase was ~0.5 log (Fig. 1, B to E).

**Fig. 2.**
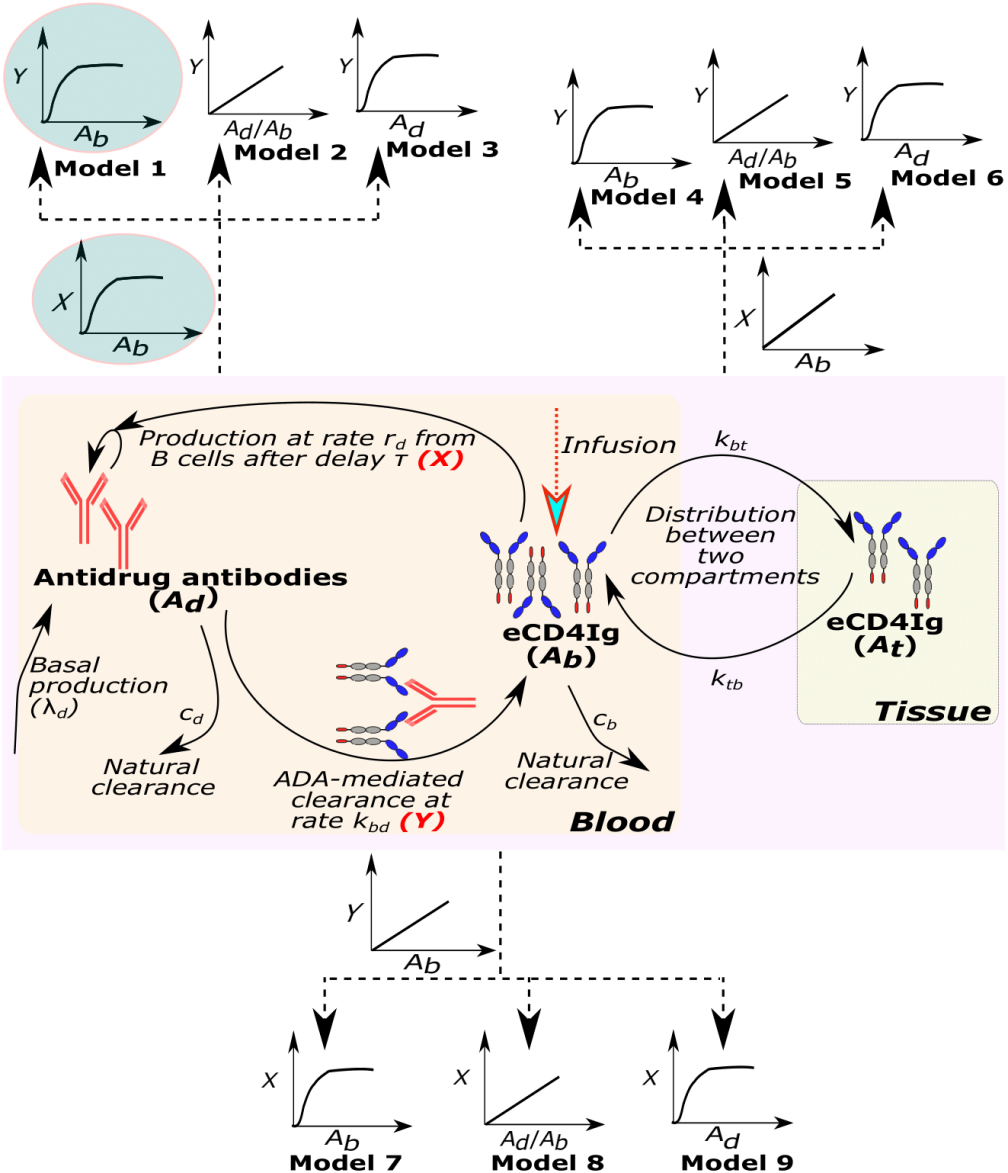
Schematic representation of the PK model to describe the dynamics of eCD4Ig in blood (*A_b_*) and ADAs in blood (*A_d_*) (pink region). In the generic version of the PK model, upon infusion, eCD4Ig in the blood moves to and from the tissue compartment at rates *k_bt_* and *k_tb_*, respectively, while naturally clearing at rate *c_b_*. In response to the presence of eCD4Ig in the blood, ADAs are produced at rate *r_d_* after a time delay of τ days that are naturally cleared at rate *c_d_* and have a basal production rate of λ*_d_*. Moreover, ADA contributes to the clearance of eCD4Ig in the blood at rate *k_bd_*. Here, we represent 9 different formulations (out of a total of 19 presented in table S2) of the mechanisms of the production of ADA in the presence of eCD4Ig (*X*) and ADA-mediated clearance of eCD4Ig (*Y*).

**Fig. 3.**
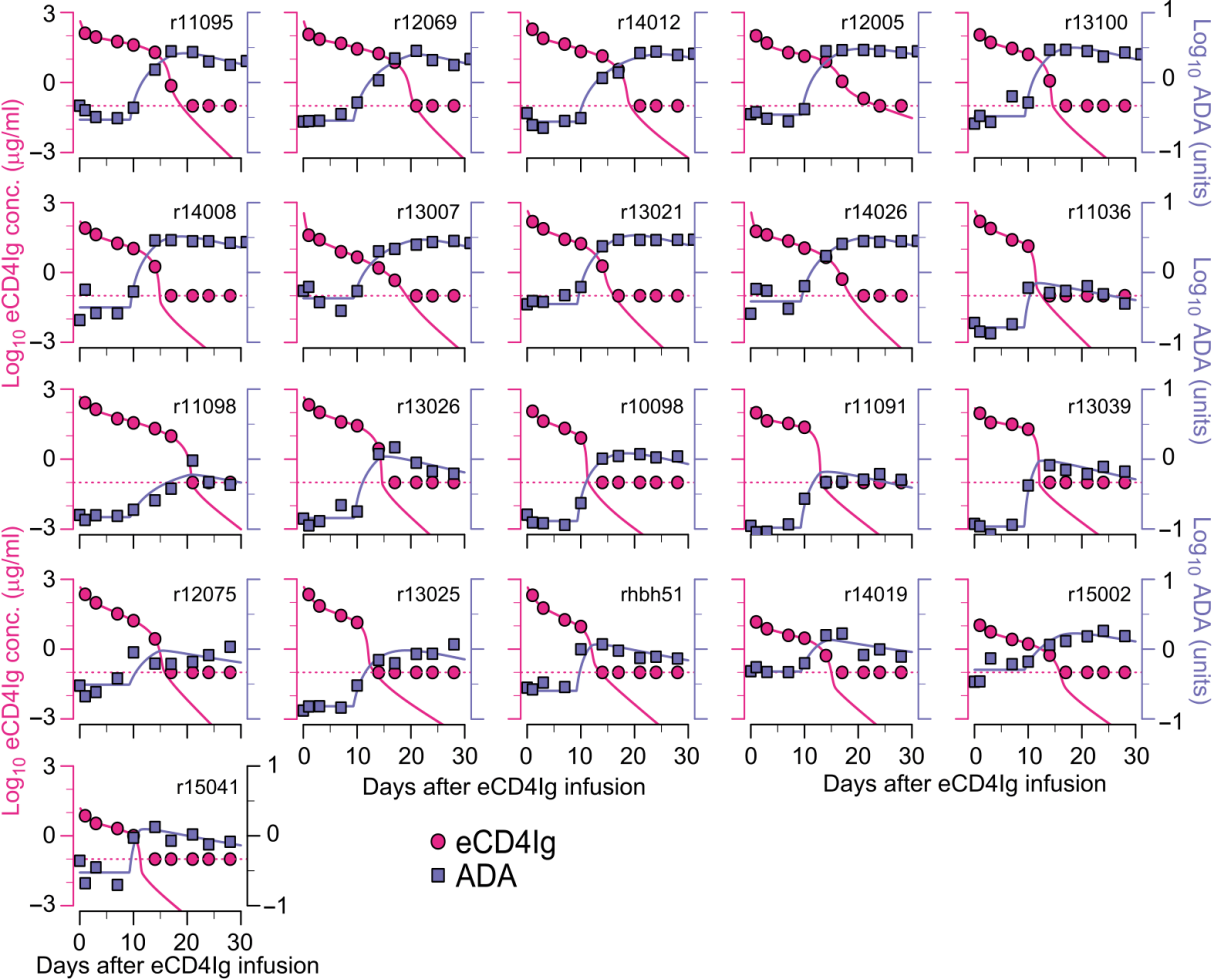
Model fits of eCD4Ig [log_10_ (μg/ml), red] and ADA [log_10_(Ab, 450 nm), blue] for 21 uninfected animals. Solid lines represent best fits of the model, while markers represent observed data. Dotted red lines represent the limit of detection of eCD4Ig (0.1 μg/ml).

To characterize the eCD4Ig and ADA dynamics in healthy RMs, we adapted a standard two-compartmental PK model to include ADA expansion and an anti-eCD4Ig effect (Methods). We compared a total of 19 PK models with different assumptions regarding the kinetics of ADA and its effect on eCD4Ig kinetics (table S2 and Fig. 2). Using model selection theory, we found that the most parsimonious model to explain the data is the one highlighted inside sky blue circles in Fig. 2 (model M9 in table S2). In this model, ADA expands with maximum rate *r* after a delay τ (*15*) and saturates depending upon eCD4Ig levels governed by the parameter *d*_50_ (*16*, *17*). Furthermore, the presence of ADA mediates the clearance of eCD4Ig with maximum rate *k_bd_* that is also saturated dependent on the eCD4Ig concentration. Individual model fits to the data are presented in Fig. 3 using the individual parameter estimates in table S3.

Because eCD4Ig infusion involved multiple doses and variants of the inhibitor among the animals, we performed a systematic search of possible covariates and correlations using the best model (table S4). We found that the data did not support any covariates for dose or variants, suggesting that the dynamics of eCD4Ig is independent of the variant and dose in the current study design (figs. S1 and S2), but further investigation is required because of the small sample size for each variant. However, positive correlations were identified between the rate of eCD4Ig transport from blood to tissue, *k_bt_*, with the elimination rate of eCD4Ig, *c_b_*, and the rate of ADA-mediated removal of eCD4Ig, *k_bd_*, with the rate of eCD4Ig-independent production of ADA, λ*_d_* [*r* = 0.65 and −0.94, respectively, using Spearman’s correlation test (table S5 and fig S3)].

Using the best model fits, we characterized the kinetics of eCD4Ig and ADA. In the absence of ADA, using population estimates in table S5, the estimated terminal half-life of eCD4Ig had a median of 5.2 days (among all variants), and the median time it takes to produce ADA after antigen recognition (τ) is estimated to be 9.3 days. The amount of eCD4Ig cleared per day was dominated by natural elimination (i.e., *c_b_A_b_*) for the first ~12 days after infusion [95% confidence interval = (10.6, 12.8) days] and afterward by ADA-mediated clearance $\left(i.e.,\frac{k_{\mathrm{bd}}A_{b}A_{d}}{\left(1+\frac{A_{b}}{b_{50}f_{b}}\right)}\right)$ (see fig. S4), driving the eCD4Ig transition from its second phase to a sharp third-phase decline (Fig. 3). Notably, ADA clears substantially even at lower eCD4Ig concentration (<10 μg/ml), as accurately reflected by the parameter value *b*_50_ (table S5 and Fig. 3). Removing the ADA-mediated clearance term results in a biphasic decline of eCD4Ig without the final fast drop (fig. S5), but a model that does not involve ADA-mediated clearance did not have statistical support (table S2). The best model fit also suggests that the production of ADA in response to eCD4Ig reaches half the maximal production rate when eCD4Ig levels in the blood are low, i.e., 0.09 μg/ml, implying that, at higher eCD4Ig levels, the expansion of ADA is maximal.

Information about ADAs is not always available, and thus, we next evaluated the impact of the absence of ADA observations on our eCD4Ig fit proceeding and estimated the same parameters (model M3 in table S2). We found that a model can recapitulate eCD4Ig kinetics in the absence of ADA observations (fig. S6), but the fits affect the estimated parameters (for example, the parameter value of the elimination rate of eCD4Ig, *c_b_*, was estimated to be 0.018/day instead of 0.37/day as in table S5). Therefore, ignoring the ADA data may affect the projected dynamics of eCD4Ig. Thus, our analysis supports fitting of ADA along with eCD4Ig, whenever sharp third-phase eCD4Ig decay is observed in the data. Last, accurately capturing ADA kinetics might play a very important role in decreasing eCD4Ig levels during multiple infusions.

### The half-life of eCD4Ig is unaffected in SHIV-infected RMs

**Fig. 4.**
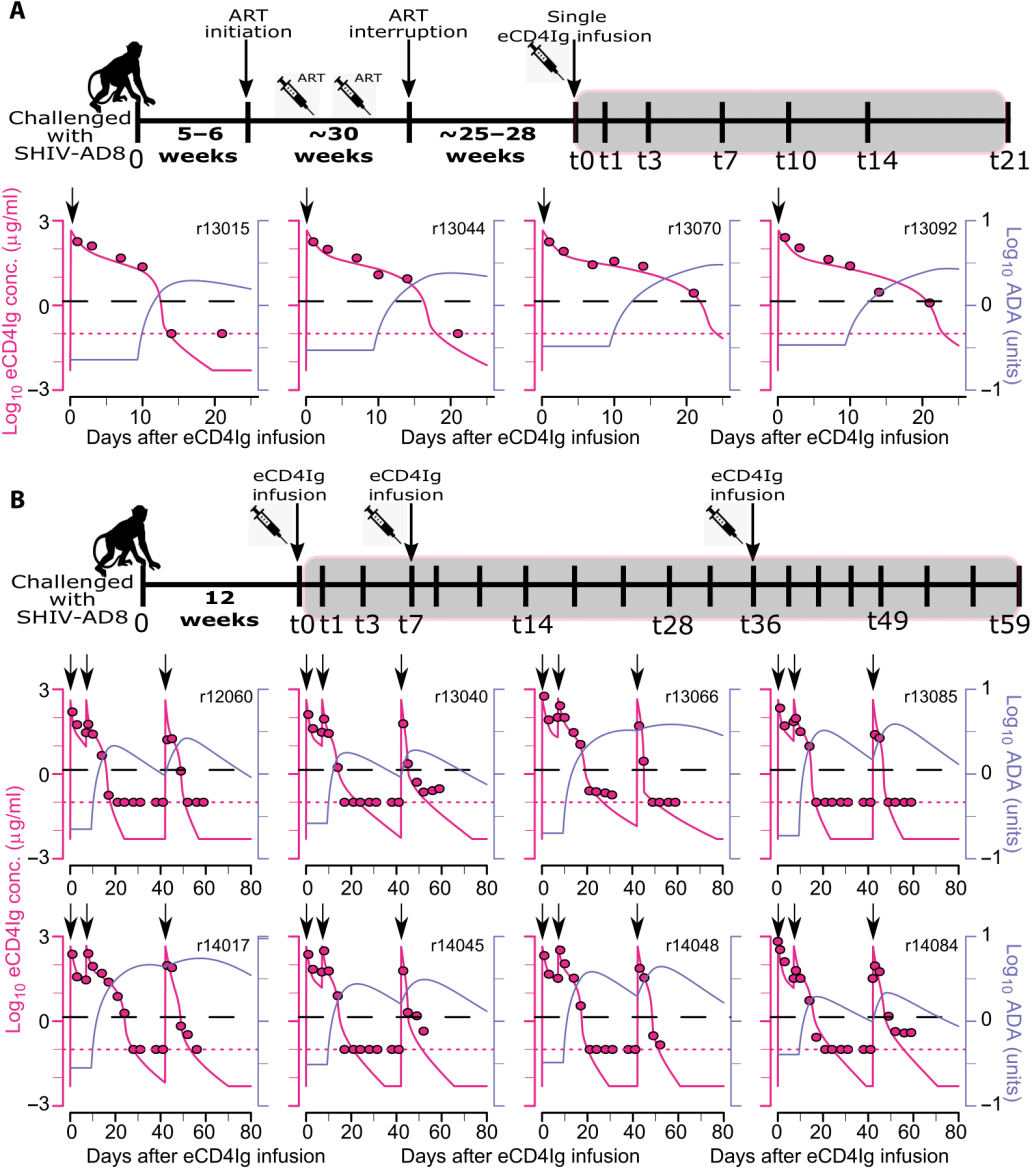
Model fits to eCD4Ig [log_10_ (μg/ml), red] and simulated ADA [log_10_ (Ab, 450 nm), blue] data from 12 infected animals. Solid lines represent best fits of the model, while markers represent observed data. Dotted red lines represent the limit of detection of eCD4Ig. (**A**) eCD4Ig dynamics in four animals that received a single dose of eCD4Ig. (**B**) eCD4Ig dynamics in eight animals that received three doses of eCD4Ig at times indicated by black arrows. Black dashed lines represent the estimated median IC_50_ of eCD4Ig that represents the concentration of eCD4Ig in serum at which we estimate that the neutralization efficacy would be 50%.

Next, we characterized the eCD4Ig PK in 12 SHIV-AD8 infected RMs. Four of these animals started ART ~4 to 5 weeks after inoculation, and after ~30 weeks, they underwent analytical treatment interruption (ATI). Approximately 25 to 28 weeks after ATI, all four animals received a single infusion of eCD4Ig (30 mg/kg) intravenously. The other eight animals did not receive ART and were given a first dose of eCD4Ig (30 mg/kg) intravenously at 12 weeks after inoculation. After receiving the first dose, they received two additional infusions (30 mg/kg) of eCD4Ig at weeks 1 and 6 after the first one. The experimental design is presented in Fig. 4 (A and B).

Because ADA was not measured in SHIV-infected RMs, we characterized the eCD4Ig PK in infected animals by simultaneously fitting eCD4Ig observations from infected animals and eCD4Ig/ADA observations from uninfected animals. We used the validated model obtained in the previous section and systematically explored for covariates of the eCD4Ig kinetic parameters in the infected group. We used estimations of the uninfected group to project the kinetics of ADA in infected animals.

We used model selection theory to compare 16 competing models with different covariates relative to the PK model parameters dependent on the infection status. The analysis revealed that having a different clearance rate of eCD4Ig in the blood (*c_b_*) and a rate of transfer of eCD4Ig between tissue and the blood compartment (*k_tb_*) for the infected animals improves the quality of fits for treated animals (ΔAIC = 17). Individual model fits to the data are presented in Fig. 4 using the individual parameter estimates in table S6. Population parameter estimates (table S7) presented that the elimination rate of eCD4Ig in plasma (*c_b_* ~ 0.68/day) was significantly higher for the infected animals compared to the uninfected (0.38/day, *P* = 0.0002, Wald test). However, the rate of transfer of eCD4Ig from tissue to the blood compartment was estimated to be slower in infected animals (*k_tb_*~ 0.25/day) than untreated animals (*k_tb_* ~ 0.50/day) (*P* = 0.0007, Wald test). These two differences yielded a terminal half-life of eCD4Ig of median 6.0 days before ADA expansion in infected animals, longer than the median half-life of 5.2 days in uninfected animals but not significantly (*P* = 0.55, Wilcoxon rank sum test).

### Mathematical model reproduces viral dynamics in SHIV-infected RMs and reveals that in vivo IC_50_ of eCD4Ig is ~25 to 30 μg/ml

Here, to quantify eCD4Ig in vivo effects including neutralizing and ADCC effects, we developed mathematical models that can recapitulate the dynamics of SHIV RNA (copies/ml) in the 12 SHIV-AD8–infected RMs for which the experimental design is presented in Fig. 4 (A and B).

**Fig. 5.**
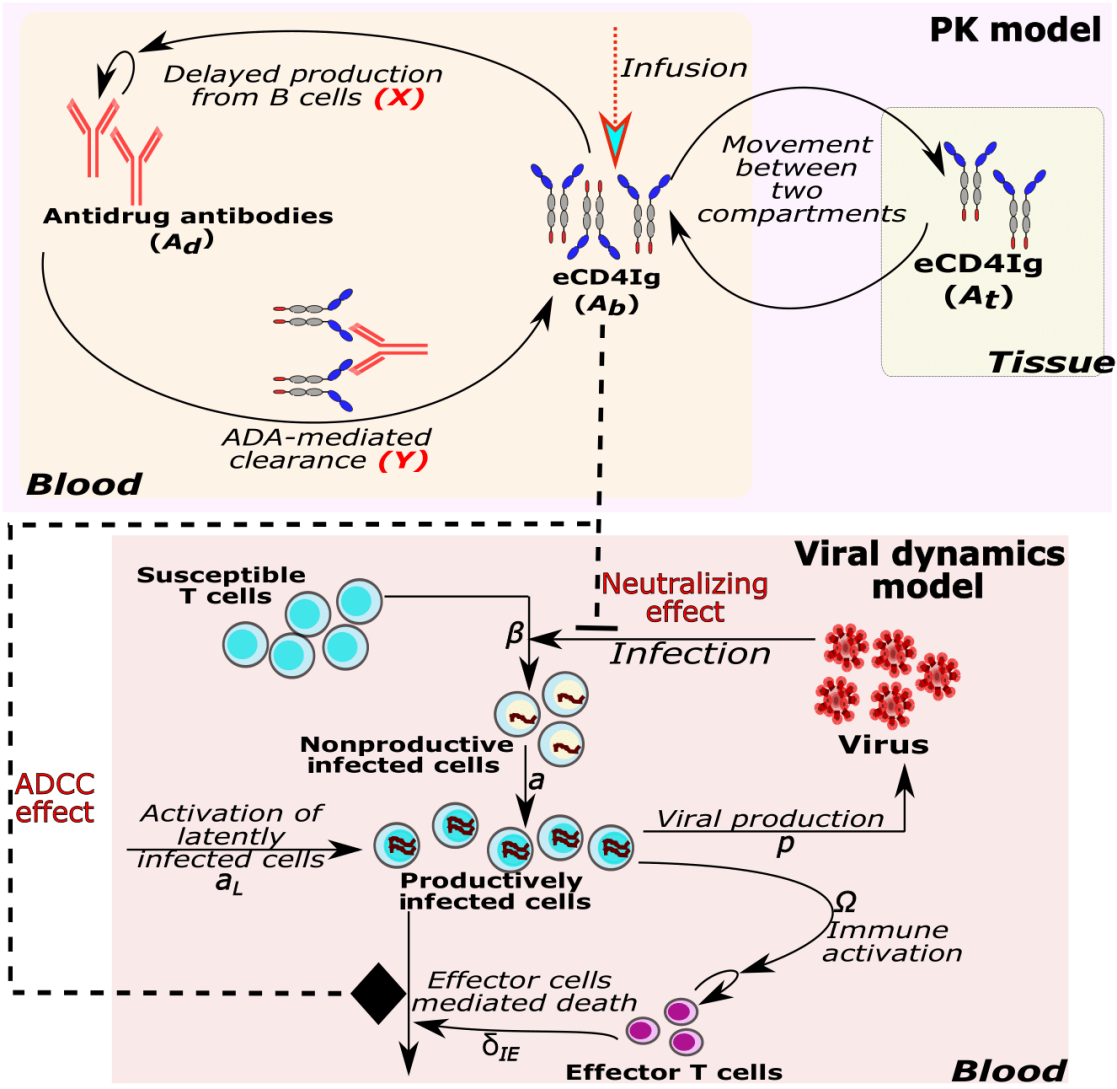
SHIV dynamics model under eCD4Ig treatment. Top: Version of the PK model best supported by the data. See Fig. 2 for more details on the description of the PK model. Bottom: Viral dynamics model. The model assumes that virus, upon introduction in the system, infects susceptible cells, converting them into infected cells at rate β. Infected cells are initially nonproductive but gradually become productively infected. These productively infected cells then produce new virions at rate *p*. The productively infected cells are also generated at rate *a_L_* from the activation of latently infected cells. The productively infected cells are naturally clear because of the cytopathic effects of the virus at rate *d_I_* (not shown in the figure) as well as because of the virus-specific effectors T cells at rate δ*_IE_* that are generated in response to the foreign antigen after activation at rate Ω. Here, we assume that eCD4Ig in the blood can affect the viral dynamics in two ways: (i) by inhibiting viral entry and thus exhibiting neutralizing effects and (ii) by enhancing the clearance of infected cells and thus exhibiting ADCC.

**Fig. 6.**
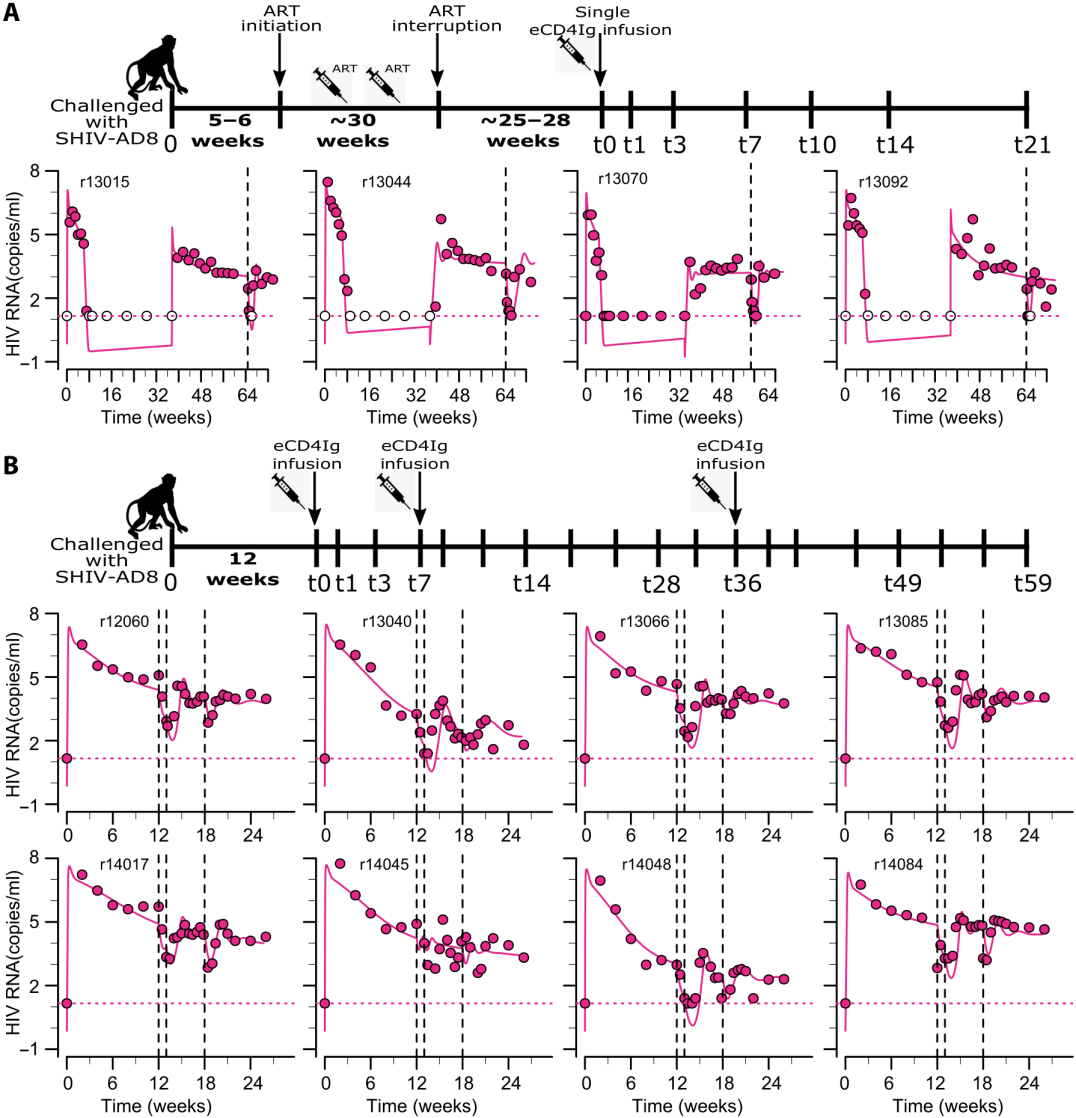
Model fits to SHIV viral dynamics during primary infection, ART, ATI and eCD4Ig passive infusions. (**A**) Fits to HIV RNA (copies/ml, red round markers) during primary infection, ART, ATI, and during eCD4Ig administration (indicated by dashed black vertical lines) in four animals. (**B**) Fits to HIV RNA (copies/ml, red round markers) during primary infection, followed by eCD4Ig administration (indicated by dashed black vertical lines) in eight animals.

We simultaneously fit competing models to SHIV RNA (copies/ml) observations from these two groups of infected RMs using the validated PK model from the previous section. The best virus dynamics model (Fig. 5) was obtained using model selection theory from a total of 11 competing models with differences in both model structures and parameter assumptions (table S8). Model fits to the observation from the two groups are shown in Fig. 6 using the individual parameters given in table S9. In the best model, susceptible cells (*T*) to SHIV-AD8 are produced from the thymus at rate λ*_T_* and become infected after coming into contact with virus (*V*). Initially, they become unproductive, infected cells (*M*) that after an eclipse phase become productively infected (*I*) and start to produce new virions. The model also considers a constant reactivation of latently infected cells adding to the pool of the productively infected cells, providing a realistic low viral level during ART that contribute to the viral rebound after ART interruption (Fig. 6A). In this model, the presence of SHIV triggers immune responses and induces effector cells (*E*), which in turn contribute to the clearance of the infected cell population.

From the best model fits, the median eCD4Ig concentration that leads to 50% inhibition (IC_50_) is estimated to be ~25 μg/ml (table S9). The model estimated only a weak ADCC-mediated effect of eCD4Ig. This was confirmed by the better quality of fits when we assumed that eCD4Ig only exhibits neutralizing effects, in comparison to when we assumed that eCD4Ig only exhibits ADCC effects (ΔAIC ~ 27). Nonetheless, this result does not necessarily imply the absence of ADCC in vivo; rather, it implies that the available data might be insufficient to detect that phenomenon.

The fitting procedure under the best model also allowed for the quantification of SHIV-AD8 viral replication parameters. In agreement with the literature for SHIV, HIV, and simian immunodeficiency virus (SIV) (*18*–*23*), our median parameter estimates for the production rate of target cells (λ*_T_*), the death rate of target cells (*d_T_*), the viral infectivity (β), the removal rate of infected cells due to effector cells (δ*_IE_*), the recruitment rate of effector cells (Ω), the infected cell population at the rate of recruitment of effector cells becomes 50% (*I*_50_), and the production rate of effector cells λ*_E_* were at 3 × 10^3^ cells/μl per day, 0.1/day (*24*), 0.9 × 10^−5^ μl virions/day, 11.0 μl/day per cell, 0.11 μl/day per cell, 39.4 cells/μl, and 1.44 × 10^−4^ cells/μl per day, respectively (*21*, *25*). Our best model further suggested that in comparison to the primary infection, after treatment interruption, both viral infectivity (β) and the removal rate of infected cells by effector cells (δ*_IE_*) increase by a factor of 9.4 and 7.8, respectively; trends were also observed in (*21*).

The model also predicts that SHIV plasma viral loads come back to set points in the long term after eCD4Ig infusion due to shrinking of eCD4Ig levels, even if IC_50_ was reduced (fig. S7). However, in the short term, there are benefits to lowering IC_50_ because it keeps the virus suppressed for a longer period (fig. S8).

### Modeling supports a role for eCD4Ig administered in the AAV vector form in the long-term remission of chronic HIV

**Fig. 7.**
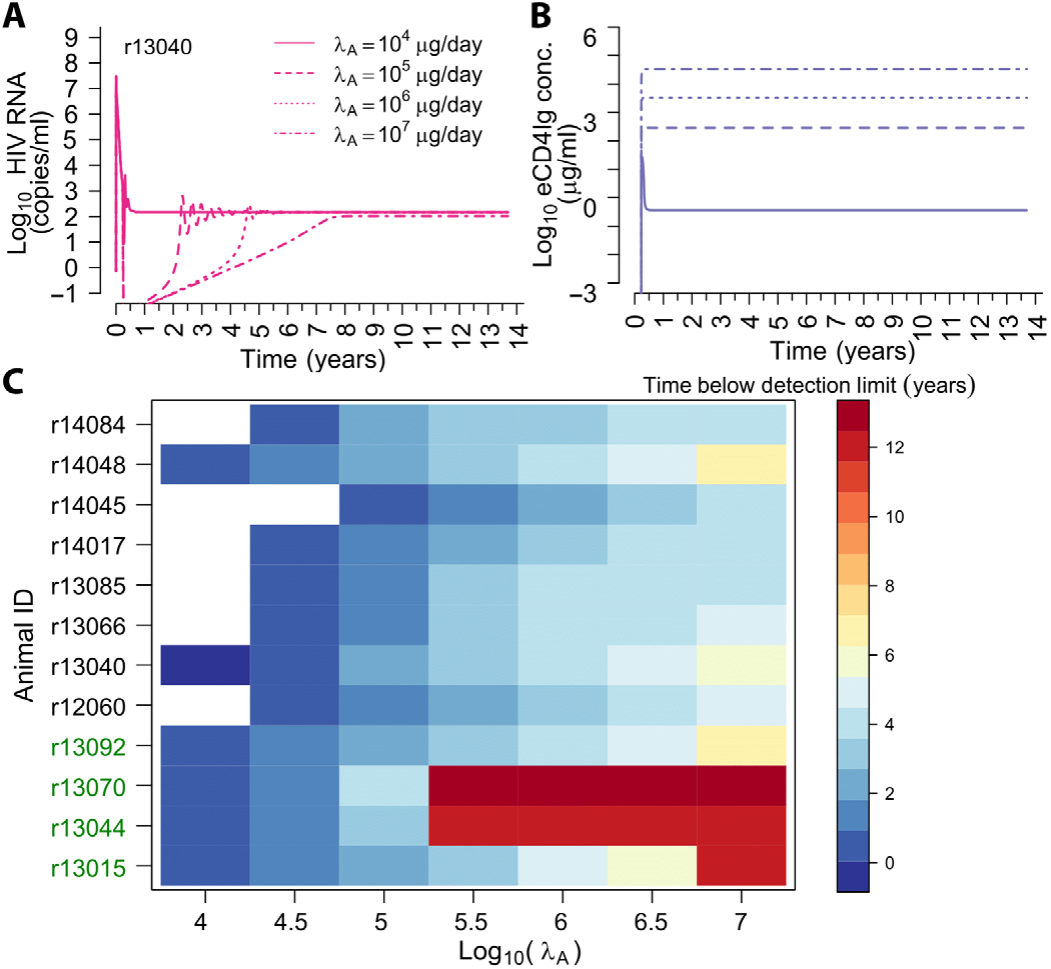
Projections of viral suppression in a scenario where eCD4Ig can be expressed continually. (**A**) HIV RNA (copies/ml) for four different continuous production levels of eCD4Ig from AAV vectors (parameter, λ*_A_*) for the animal r12060. (**B**) Corresponding eCD4Ig levels and (**C**) number of years for which the virus was suppressed in simulations for 12 animals for different values of λ*_A_*.

We further investigated whether the continual expression of eCD4Ig, for example, using an AAV vector, would be able to overcome ADA-related limitations and allow for a persistent suppression of viremia. We simulated such scenario by replacing the equation$\frac{dA_{b}}{\mathrm{dt}}=-k_{\mathrm{bt}}A_{b}+k_{\mathrm{tb}}A_{t}-Y(t)-c_{b}A_{b}$ in the original system by $\frac{dA_{b}}{\mathrm{dt}}=\lambda_{A}-k_{\mathrm{bt}}A_{b}+k_{\mathrm{tb}}A_{t}-Y(t)-c_{b}A_{b}$, where we assumed λ*_A_* is the constant AAV-mediated eCD4Ig production rate in this animal model. Simulations suggest that production rates upward of 10^5^ μg/day are sufficient to overcome ADA-mediated effects on eCD4Ig, thus allowing for persistent viral suppression for years (Fig. 7). In contrast, if the production rate of eCD4Ig is lower than 10^4^ μg/day, then the system will exhibit no viral suppression.

**Fig. 8.**
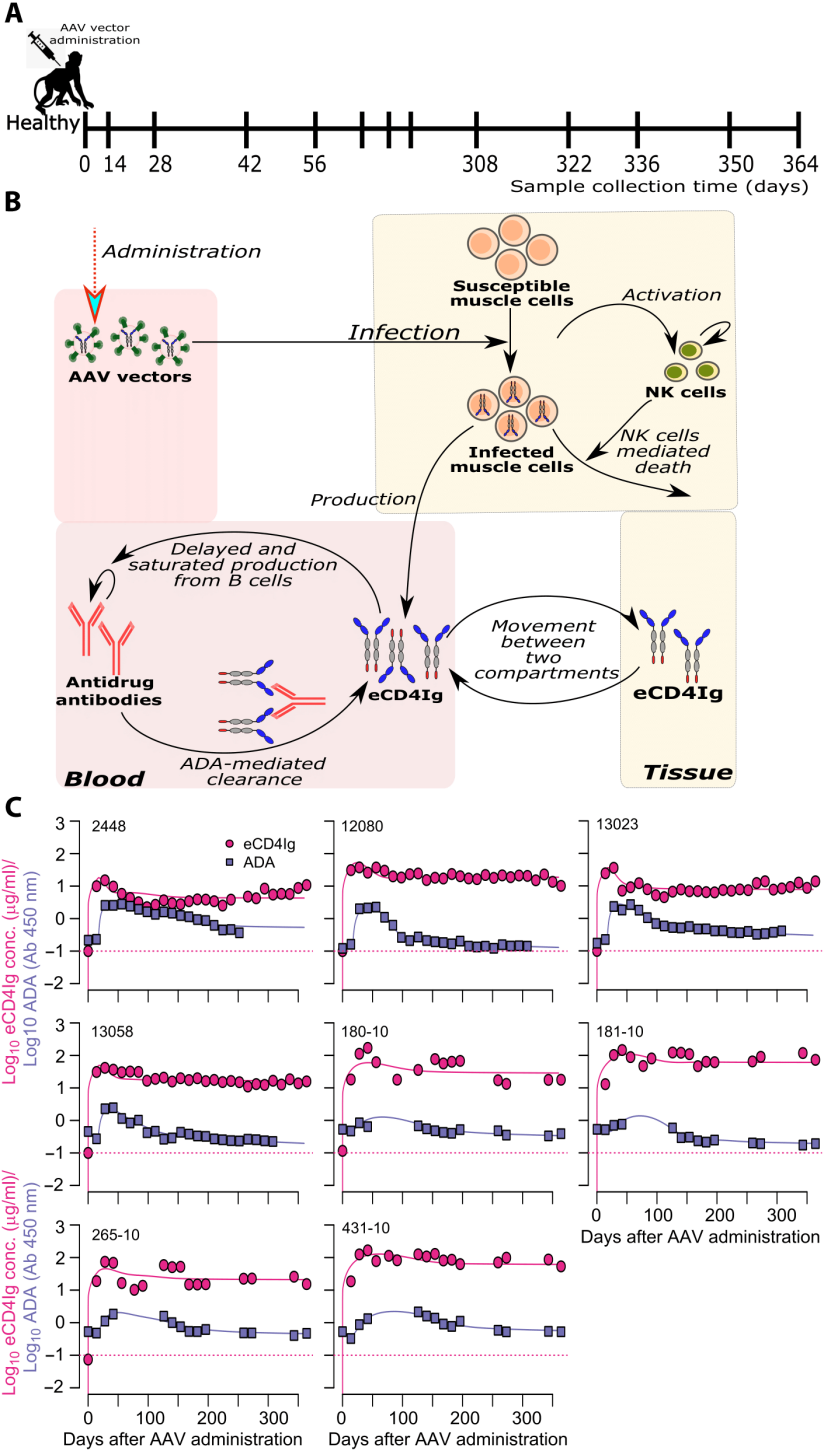
PKs of eCD4Ig and ADA after administration of eCD4Ig in AAV vector form in healthy (uninfected) RMs. (**A**) Experimental procedure. (**B**) Schematic version of the mathematical model that best recapitulates the observed data: The model assumes that AAV vectors, upon introduction in the system, transduce susceptible muscle cells, converting them into transduced muscle cells. The production of eCD4Ig from transduced muscle cells induces ADA in a delayed and saturating manner. The ADA naturally binds to eCD4Ig, enhancing its clearance. In the model, the transduction of muscle cells with AAV vectors also induces immune responses such as NK cells that then clear them (*26*–*29*). (**C**) Model fits to eCD4Ig [log_10_ (μg/ml), red] and simulated ADA [log_10_(Ab, 450 nm), blue] data from eight uninfected animals. Solid lines represent best fits of the model, while markers represent observed data. Dotted red lines represent the limit of detection of eCD4Ig.

To further confirm that eCD4Ig administered in the AAV vector form can allow for persistent viral suppression by producing eCD4Ig at levels greater than 10^4^ μg/day, we analyzed the kinetics of eCD4Ig in the presence of ADA from eight healthy RMs after administering a single shot of 2.0 × 10^13^ AAV vectored particles encoding eCD4Ig and 0.5 × 10^13^ AAV vectored particles encoding tyrosylprotein sulfotransferase 2 (*TPST2*) to ensure sulfation of the coreceptor-mimetic peptide on eCD4Ig (Fig. 8A) (*7*, *10*, *12*). To do that, we developed mathematical models of AAV-mediated production of eCD4Ig (table S11) and fit them to AAV-mediated eCD4Ig levels and ADA. Notice that this model only studies the AAV vector and eCD4Ig dynamics and is uncoupled from the virus dynamics presented before. We performed these analyses with the caveat that AAV produces the rhesus version of eCD4-Ig (RM CD4 domains 1 and 2 with the RM IgG2 Fc), but the protein infused in the RMs studied in the previous sections were human-based, which may be a confounding factor. Using model selection theory, we found a parsimonious model (Fig. 8B) from a total of six competing models with different mechanisms for eCD4Ig production (table S11). Model fits to eCD4Ig and ADA observations are shown in Fig. 8C using the individual parameters given in table S12. In the best model, AAV vectors transduce susceptible cells that can in turn produce eCD4Ig. Following eCD4Ig production, it is assumed that eCD4Ig induces ADA production and ADA enhances clearance of eCD4Ig in a similar fashion as to what was proposed in previous sections (Fig. 2). Furthermore, the model additionally considers the activation of immune cells such as natural killer (NK) cells to clear AAV-transduced cells (*26*–*29*). At the steady state, our model projects that the median λ*_A_* is upward of 10^4.8^ μg/day (minimum = 10^4.5^, maximum = 10^5.3^), suggesting that eCD4Ig administered in the AAV vector form might allow the long-term remission of chronic HIV.

## DISCUSSION

In this study, we introduce a mathematical model to understand the PK of eCD4Ig in the presence of ADA response and the in vivo pharmacodynamics (PD) of eCD4Ig against SHIV by fitting differential equation models to serum eCD4Ig, ADA, and plasma SHIV-RNA levels from healthy and SHIV-AD8–infected RMs after passive infusion(s) of eCD4Ig. We systematically performed model selection by fitting 19 models for the PK of eCD4Ig explicitly including mechanisms for ADA response and by fitting 11 models to SHIV viral load in the absence and presence of eCD4Ig. We arrived at a set of differential equations that mechanistically characterizes (i) the eCD4Ig clearance kinetics in the presence and absence of ADA, and in the presence and absence of SHIV-infection; (ii) the timing and speed of ADA response; and (iii) the in vivo PD of eCD4Ig against SHIV-AD8 in terms of the IC_50_. Using this model, we were able to project the necessary conditions of eCD4Ig production that predicts long-term remission of SHIV viremia in the absence of ART.

From our modeling, we found that the PK of eCD4Ig and ADA response did not differ significantly among eCD4Ig variants. We found that before the ADA response, eCD4Ig elimination rate constant was about twofold lower for SHIV-AD8–infected RMs than healthy animals; however, the median terminal half-life of eCD4Ig was estimated to be comparable in the range of ~5 to 6 days in both groups. Because we did not find a significant correlation between the elimination rate constant and the transport rate from tissue to blood in the best model, further studies would be needed to understand the mechanistic explanation of these differences in the eCD4Ig PK between infected and uninfected animals. We also found that ADA response takes about 9 days to start expanding, leading to a drastic drop in eCD4Ig levels about 12 days after infusion. This delay in ADA response concurs with PK literature for other high–molecular weight compounds (*15*, *17*, *30*, *31*). On the other hand, despite the high clearance mediated by ADA, ADA expansion saturates rather rapidly for high values of eCD4Ig, which could allow for a limited impact of eCD4Ig clearance if it can be produced continually as in the case of AAV vectors. We found that in the absence of ADA observations, these kinetics parameters may have different and less realistic estimated values. Despite the potential of eCD4Ig as a therapeutic approach against SHIV, its life span is substantially shorter than isolated broadly neutralizing antibodies in this type of animal models (*32*–*34*).

We then used plasma viral load levels from SHIV-AD8–infected RMs receiving eCD4Ig in two different stages of infection to characterize its in vivo effect against SHIV: (i) late during infection, after animals had started and stopped ART, and (ii) early during infection after 12 weeks of challenge. To reduce biases on the in vivo effect of eCD4Ig, we looked first for the best model that recapitulates the viral kinetics in the absence of eCD4Ig. From our best model, we found an in vivo IC_50_ of ~25 μg/ml that did not differ significantly between the two groups of animals receiving this inhibitor. The estimated in vivo IC_50_ of eCD4Ig is ~100-fold higher than in vitro estimates of <0.05 μg/ml and in vitro IC_50_ estimates of other bNAbs (*7*, *9*), due to possibly different physiological processes that affect neutralization. This phenomenon is not unique to this inhibitor and has been observed in other scenarios for HIV and for small molecular agents targeting other viruses (*25*, *35*, *36*).

One of the main attributes of eCD4Ig in vitro is its ADCC ability (*11*). Although ADCC may be relatively unimportant in terms of killing recently infected cells after eCD4Ig infusion, a faster virus decline after its administration might reveal that this protein is able to mediate this cytotoxic mechanism (*37*). In our study, we compared mathematical models with and without ADCC but found better support for a model without ADCC. Even our model that included ADCC predicts an insignificant increase in the death rate of infected cells. However, this result is not definitive that eCD4Ig does not mediate ADCC in vivo but may reflect that viral load sampling during the eCD4Ig infusion was not frequent enough to differentiate such phenomena. If present, ADCC could serve an important role in eliminating the long-term latent reservoir; however, further experiments are required to investigate this.

One characteristic of our model is that it does not include effector cell exhaustion. In the literature, mathematical models have also considered saturated elimination of effector cells representing CD8^+^ T cell exhaustion (*38*, *39*). Adding exhaustion in this way results in bistability of the virus dynamics—i.e., the possibility of having multiple set points, long-term ART-free virus remission, or virus rebound with a fixed set of parameters (*38*, *39*). However, our model does not consider CD8^+^ T cell exhaustion because (i) it will require the estimation of more parameters that would not be identifiable; (ii) exhaustion has been used to explain posttreatment controllers, which was not the case for the analyzed viral loads in this study; and (iii) models without exhaustion have also fit well viral rebound data in the past.

AAV-based production of eCD4Ig has been shown to protect against SHIV and SIV infection in RMs and lower the viral set point in SIV-infected RMs (*7*, *10*). and AAV vectors allow the constant production of the inhibitor after transducing muscle cells that can continually manufacture eCD4Ig. This technology overcomes the challenge posed by the shorter half-life of eCD4Ig and also opens a possibility of using it as means of prevention and also as a therapeutic approach for HIV cure. We adapted our model to project the plasma viral load in the SHIV-infected macaques in the absence of ART if eCD4Ig starts to be produced constantly. Using our validated model that takes into account eCD4Ig PK/PD and the ADA response, we found that it is only possible to obtain a long-term remission of viremia in this animal model if there is a constant production of eCD4Ig of 10^5^ μg/day in all animals. A limitation of this modeling approach is that it assumes constant production, when it has been shown that eCD4Ig production can fluctuate over time. However, we believe that in the long term, eCD4Ig fluctuations might be reduced (*7*, *10*). Another limitation is that we fit models to AAV-based eCD4Ig that produces the rhesus version of eCD4-Ig, but the threshold of virus production was based from model fits to eCD4Ig variants that were human-based.

Our results are somewhat limited by a small sample size of 21 healthy and 12 SHIV-infected animals. This resulted in some limitation in our modeling, such as fixing some virus kinetics parameters based on estimates from other animal models or estimates from models of HIV. However, we found that, for each animal, the sampling was frequent enough to select a mathematical model among a list of competing ones with reasonable mechanistic assumptions. On the other hand, the data might have not been sampled frequent enough to differentiate models that require parameters with faster kinetics, such as the death rate of infected cells. Therefore, models that include eCD4Ig mediated killing of infected cells, such that those that included ADCC were not favored.

In summary, our mathematical model can recapitulate the PK of eCD4Ig, the ADA response against this inhibitor, the viral kinetics of SHIV-AD8 in infected RMs, and the in vivo neutralization effect of eCD4Ig in this SHIV animal model. Our model is also able to project conditions for ART-free long-term remission of SHIV viremia for the case when eCD4Ig is produced constantly. This study continues a tradition of illustrating the capacity of mathematical models to characterize virus dynamics and the in vivo therapeutic efficacy.

## MATERIALS AND METHODS

### Experimental data

#### Experimental data from healthy RMs receiving infused eCD4Ig

Twenty-one uninfected RMs were administered with four variants of eCD4Ig and three different doses, namely, eCD4IgG1-v34-LS, eCD4IgG1-v34, eCD4IgG2-v26-LS, and eCD4IgG2-v26 (Fig. 1). Nine animals in groups of three received eCD4IgG1-v34-LS in three different doses at 30, 10, and 1 mg/kg, three animals received eCD4IgG1-v34 at 30 mg/kg, three animals received eCD4IgG2-v26-LS at 30 mg/kg, three animals received eCD4IgG2-v26-LS at 10 mg/kg, and three animals received eCD4IgG2-v26 at 30 mg/kg. The concentration of eCD4Ig and ADAs was measured at days 1, 3, 7, 10, 14, 17, 21, 24, 28, and 31. Versions v34 for eCD4IgG1 and v26 for eCD4IgG2 were optimized versions of the wild-type Fc sequences for thermostability and manufacturability. The LS variants contained the M428L/N434S amino acid substitutions in the IgG1 or IgG2 Fc that have been shown to increase the half-life of antibodies.

#### Experimental data from SHIV-infected RMs

We analyzed data also from two groups of RMs that were intravenously challenged with SHIV-AD8 but received eCD4Ig in different ways. A first group (*n* = 4) received ART therapy between weeks 5 and 6 after infection and stayed on ART for about 30 weeks by which the treatment was interrupted. After 176 to 190 days on treatment interruption, RMs received a single infusion of eCD4Ig (30 mg/kg) intravenously (two received eCD4IgG1-v34-LS and the other two received eCD4Ig2-v26-LS). A second group (*n* = 8) was infected with SHIV-AD8 intravenously, did not receive any ART, and directly were given a first dose of eCD4IgG1-v34-LS (30 mg/kg) intravenously at 84 days after inoculation. After receiving the first dose, they received two additional infusions (30 mg/kg) of eCD4Ig at weeks 1 and 6 after the first one. Four animals received eCD4IgG1-v34-LS as their second infusion, the other four received eCD4IgG2-v26-LS. All eight animals received eCD4IgG2-v26-LS for their third infusion.

The viral loads (RNA copies/ml) were measured weekly, weekly, and biweekly during the primary infection, on ART until viral loads become undetectable, and during ATI, respectively, in the first group. After eCD4Ig infusion, the viral load measurements were done at days 1, 3, 7, 10, 14, 21, 35, 49, and 63 (day 49 was missing for two monkeys), whereas eCD4Ig (μg/ml) was measured at days 1, 3, 7, 10, 14, and 21. In the second group, the viral load measurements were less frequent (biweekly) during the primary infection measured; however, after eCD4Ig infusion, they were measured frequently at days 0, 3, 7, 8, 14, 17, 21, 24, 28, 31, 35, 38, 41, 45, 49, 52, 56, 59, 63, 70, 84, and 98. Similarly, eCD4Ig was measured at days 1, 3, 7, 8, 10, 14, 17, 21, 24, 28, 31, 38, 41, 43, 45, 49, 52, 56, and 59 after the first infusion.

#### Experimental data from healthy RMs receiving AAV vectors

Eight uninfected RMs received a single shot of 2.0 × 10^13^ AAV vectored particles encoding eCD4Ig and 0.5 × 10^13^ AAV vectored particles encoding *TPST2* (*7*, *10*). Thereafter, the concentration of eCD4Ig and ADAs was measured biweekly until week 52.

### Animal welfare

The data used in this work were collected in strict accordance with the recommendations in the *Guide for the Care and Use of Laboratory Animals* of the National Institutes of Health (NIH). The study protocol was approved by the Institutional Animal Care and Use Committees of the Wisconsin National Primate Research Center with protocol number G005045.

### Mathematical modeling

#### PK model of eCD4Ig and ADA response after single or multiple eCD4Ig infusions

We adapted a standard two-compartmental PK model (*40*) to compare a wide variety of models for the anti–eCD4Ig-ADA kinetics and effect on eCD4Ig clearance (Fig. 1B and detailed description in table S2). In the standard two-compartmental model, *A_b_* and *A_t_* represent the amount of eCD4Ig in blood and tissue (or some other compartment), respectively. The eCD4Ig is distributed from blood to tissue and back at rates *k_bt_* and *k_tb_*, respectively, and is cleared from the blood in the absence of ADA at rate *c_b_*. In the model, *A_d_* represents the plasma levels of anti–eCD4Ig-drug antibodies (ADA) with a basal production with rate λ*_d_* and a clearance rate of *c_d_*. The model assumes that ADA is produced mainly as a response to the levels of eCD4Ig (*17*). We explore different possibilities of the production of ADA in response to ADA that is represented by the function *X*(*t*). Upon production, we also explored how ADA mediates eCD4Ig clearance in the blood using the function *Y*(*t*). Following these assumptions, the model has the form

$$\frac{dA_{b}}{\mathrm{dt}}=-k_{\mathrm{bt}}A_{b}+k_{\mathrm{tb}}A_{t}-Y(t)-c_{b}A_{b}$$

$$\frac{dA_{t}}{\mathrm{dt}}=k_{\mathrm{bt}}A_{b}-k_{\mathrm{tb}}A_{t}$$

$$\frac{dA_{d}}{\mathrm{dt}}=\lambda_{d}+X(t)-c_{d}A_{d}$$

The different competing forms of *X*(*t*) and *Y*(*t*) are described in detail in table S2. Note that in this model, in the absence of ADA, the terminal half-life of eCD4Ig is given by the equation $t_{1/2}=\frac{\text{ln}(2)}{\frac{1}{2}[(k_{\mathrm{bt}}+k_{\mathrm{tb}}+c_{b)}-\sqrt{{\left(k_{\mathrm{bt}}+k_{\mathrm{tb}}+c_{b}\right)}^{2}-4k_{\mathrm{tb}}c_{b}}]}$ (*41*).

#### Viral dynamics model

We developed mathematical models for SHIV-AD8 kinetics in RMs by doing adaptations to the basic model of virus dynamics (*42*–*44*). This model assumes that target cells (possibly a subset of CD4^+^ T cells) (*T*) are produced from thymus at rate λ*_T_* and naturally cleared at rate *d_T_*. After coming into contact with virus (*V*) at rate β, target cells become initially unproductive (*M*) cells with the same death rate. Unproductive infected cells become productively infected (*I*) after an eclipse phase of 1/*a* days. These infected cells produce new virus at rate *p* and the virus clears out at rate *c*. We consider that latently infected cells reactivate with constant rare *a_L_*. We assumed that the presence of HIV triggers cytolytic immune responses and induces the proliferation of effector cells (*E*) at rate Ω. We further assumed the impairment of effectors T cells by assuming a saturating effect of infected cells on the induction of effector cells with parameter *I*_50_ as high viral loads (and therefore high infected cell densities) are thought to induce immune exhaustion (*39*). These effector cells contribute to the clearance of infected cell population at rate δ*_IE_* on top of the natural clearance of infected cells at rate *d_I_* due to the cytopathic nature of the virus. In the absence of antigen, effector cells are produced at rate λ*_E_* and cleared at rate *d_E_*. The viral dynamics model is thus governed by the set of differential equation as given below

$$\frac{\mathrm{dT}}{\mathrm{dt}}=\lambda_{T}-d_{T}T-\beta \text{VT}$$

$$\frac{\mathrm{dM}}{\mathrm{dt}}=\beta \mathrm{VT}-\mathrm{aM}-d_{T}M$$

$$\frac{\mathrm{dI}}{\mathrm{dt}}=a_{L}+\mathrm{aM}-d_{I}I-\delta_{\mathrm{IE}}\mathrm{IE}$$

$$\frac{\mathrm{dV}}{\mathrm{dt}}=\mathrm{pI}-\mathrm{cV}$$

$$\frac{\mathrm{dE}}{\mathrm{dt}}=\lambda_{E}+\frac{\Omega \mathrm{IE}}{I+I_{50}}-d_{E}E$$

We explored competing models as a specific case of this model and with different assumptions of these parameters when fitting to the viral load data.

#### Modeling ART and eCD4Ig treatment

During ART, we modify β*_ART_* = (*1* − ϵ*_ART_*)β in the viral dynamics model because ART is well known to efficiently reduce the infectivity of the virus with efficacy ϵ*_ART_* (*42*), whereas after treatment interruption (ATI), we have β*_ATI_* = β^*^β and modify $\delta_{\mathrm{IE}}=\delta_{\mathrm{IE}}^{*}\delta_{\mathrm{IE}}$, where β^*^ and $\delta_{\mathrm{IE}}^{*}$ are changes in the viral infectivity and the removal rate of infected cells by effector cells during ATI in comparison to the primary infection (*21*). On the other hand, the effect of eCD4Ig on the viral life cycle is currently unknown. Because eCD4Ig drug concentration data are available, we explore such effects of eCD4Ig by assuming that eCD4Ig blocks viral entry and enhances infected cell killing (ADCC effect). This is modeled by replacing β by $\beta (\frac{1}{1+\frac{A_{b}}{f_{b}{\text{EC}}_{50}}})$ and *d_I_* by$\;d_{I}(1+\frac{\phi A_{b}}{f_{b}})$ after eCD4Ig infusion, where EC_50_ is the in vivo IC_50_ of eCD4Ig (the concentration of eCD4Ig in the blood at which neutralization efficacy is 50%) and ϕ is the factor by which eCD4Ig enhances killing of infected cells.

#### PK model of eCD4Ig and ADA response from eCD4Ig administration in the form of AAV vector

We modified our previous PK model to recapitulate eCD4Ig and ADA after eCD4Ig administration in the form of AAV vector (Fig. 8B and detailed description in table S10). From the previous PK model, we have *A_b_* and *A_t_* representing the amount of eCD4Ig in blood and tissue (or some other compartment), respectively. Similarly, *A_d_* represents the amount of ADAs in the blood. Among these models, the movement of eCD4Ig between the blood and the tissue compartment is modeled through rates *k_bt_* and *k_tb_*, whereas eCD4Ig is cleared from the blood at rate *c_b_*. There is also a background production rate of ADA at rate λ*_d_* and a clearance rate of *c_d_*. There is also an on-off switch denoted by *r*(*t*) that controls the production of ADA in response to eCD4Ig in a linear fashion that gets saturated by the amount of eCD4Ig (*d*_50_). Last, ADA mediates clearance of eCD4Ig antibodies (*k_bd_*), and this clearance rate gets saturated as eCD4Ig in the blood increase (*b*_50_). The new components in this model include (i) the number of AAV vectors administered (*V_AAV_*), (ii) the number of target muscle cells that become transduced with AAV vectors (*I_AAV_*), and (iii) the transient immune response mounted potentially in the form of NK cells against muscle cells transduced with AAV vectors (*E_NK_*). The target muscle cells (*T_AAV_*) that are assumed to remain constant (due to a short half-life of AAV vectors) become transduced with AAV vector at rate β*_AAV_*. The turnover rates of AAV vectors and susceptible muscle cells are denoted by *d_AAV_* and δ*_AAV_*, respectively, whereas cells transduced by helper viruses such as adenoviruses likely die at an additional rate of δ*_N_* as a result of AAV replication (*45*) and due to the immune responses such as NK cells elicited by AAV vectors (*26*–*29*). Moreover, we assume that AAV vector–transduced muscle cells produce eCD4Ig at rate *p_AAV_*. While there is a background production rate of immune response such as NK cells at rate λ*_N_* and a clearance rate of *c_N_* in the absence of AAV vectors, the presence of AAV facilitates the expansion of such responses at a rate ζ, which is assumed to get saturated at high numbers of AAV vector–transduced muscle cells (controlled by the parameter *n*_50_). The last piece of new additions in the model includes exhaustion of ADA production [i.e., $\frac{s(t)A_{b}}{\left(e_{50}f_{b}+A_{b}\right)}$]. The inclusion of this term was essential to recapitulate transient ADA dynamics observed in some of the animals, although eCD4Ig levels remained at higher levels in those animals (Fig. 2 and table S10). Following these assumptions, the model has the form

$$\frac{dV_{\mathrm{AAV}}}{\mathrm{dt}}=-\beta_{\mathrm{AAV}}V_{\mathrm{AAV}}T_{\mathrm{AAV}}-d_{\mathrm{AAV}}V_{\mathrm{AAV}}$$

$$\frac{dI_{\mathrm{AAV}}}{\mathrm{dt}}=\beta_{\mathrm{AAV}}V_{\mathrm{AAV}}T_{\mathrm{AAV}}-\delta_{\mathrm{AAV}}I_{\mathrm{AAV}}-\delta_{N}E_{\mathrm{NK}}I_{\mathrm{AAV}}$$

$$\frac{dA_{b}}{\mathrm{dt}}=p_{\mathrm{AAV}}I_{\mathrm{AAV}}-k_{\mathrm{bt}}A_{b}+k_{\mathrm{tb}}A_{t}-\frac{k_{\mathrm{bd}}A_{b}A_{d}}{\left(1+\frac{A_{b}}{b_{50}f_{b}}\right)}-c_{b}A_{b}$$

$$\frac{dA_{t}}{\mathrm{dt}}=k_{\mathrm{bt}}A_{b}-k_{\mathrm{tb}}A_{t}$$

$$\frac{dA_{d}}{\mathrm{dt}}=\lambda_{d}+\frac{r(t)A_{b}}{\left(d_{50}f_{b}+A_{b}\right)}-\frac{s(t)A_{b}}{\left(e_{50}f_{b}+A_{b}\right)}-c_{d}A_{d}$$

$$\frac{dE_{\mathrm{NK}}}{\mathrm{dt}}=\lambda_{N}+\frac{\zeta E_{\mathrm{NK}}I_{\mathrm{AAV}}}{\left(n_{50}+I_{\mathrm{AAV}}\right)}-c_{N}E_{\mathrm{NK}}$$

where *r*(*t*) = 0 if *t* < τ but *r*(*t*) = *r_d_* if *t* ≥ τ and *s*(*t*) = 0 if *t* < τ but *s*(*t*) = *s_d_* if *t* ≥ τ. Notice that this model aims to analyze eCD4Ig and ADA kinetics in the AAV vector infusion context and is decoupled from previous virus dynamics modeling. Furthermore, it is to be noted that the above model could theoretically exhibit bistability because of the presence of saturated activation and elimination in the equation for *A_d_* (*38*, *39*). Because the goal of this model was to determine whether it is possible for the developed AAV technologies to produce eCD4Ig over levels for HIV remission, we did not explore the theoretical bistability here.

### Fitting procedure

We used a nonlinear mixed effects modeling approach to perform model fitting (*46*). In this approach, the plasma viral load, the serum eCD4Ig, and the serum ADA observations for animal *i* at time *j* were modeled as log_10_ *y_ij_* = *V*(*t_j_*; θ*_i_*) + ϵ*_V_*, log_10_ *z_ij_* = *A*(*t_j_*; θ*_i_*) + ϵ*_A_*, and log_10_ *w_ij_* = *B*(*t_j_*; θ*_i_*) + ϵ*_B_*, respectively, with $\epsilon_{V}\sim N(0,\sigma_{v}^{2})$, $\epsilon_{A}\sim N(0,\sigma_{a}^{2})$, and $\epsilon_{B}\sim N(0,\sigma_{b}^{2})$ being the measurement errors for the logged viral load, logged serum eCD4Ig concentration, and logged serum ADA levels, respectively. Here, *V*(*t_j_*; θ*_i_*), *A*(*t_j_*; θ*_i_*), and *B*(*t_j_*; θ*_i_*) are the solution of the model for variables representing the viral load, eCD4Ig, and ADA levels using the animal specific parameter set θ*_i_*. We assumed that each parameter in θ*_i_* is drawn from a population distribution. The distribution of each parameter has fixed effect $\bar{\theta }$ or the median value over the population of animals, and the random effects η*_i_* represent its variability in the population, assumed to be $\eta_{j}\sim N(0,\sigma_{\theta }^{2})$ with SD σ_θ_. When random effects of the parameters are not independent, the vector of random effects follows a multinormal distribution: η ~ N(0, Ω), with Ω as the variance-covariance matrix based on the values σ_θ_ and correlations between the individual parameters. We used the software Monolix (www.lixoft.eu) to implement this modeling framework. Monolix uses the stochastic approximation of the expectation-maximization algorithm to obtain the maximum likelihood estimation of the population parameter vector $\bar{\theta }$, SDs of random effects σ_θ_ (or the matrix Ω if the model includes correlations), and the measurement errors σ*_v_*, σ*_a_* and σ*_b_*.

Last, for each model, Monolix also computes the Akaike information criteria (AIC = −2 log *L* + 2*m*, where *m* is the number of parameters estimated and *L* is the maximum likelihood of the model) for model selection. We determined the support for one model over another using model selection theory based on AIC. We assumed that a model has similar support from the data if the difference between its AIC and the best model (lowest) AIC is less than 2 (*47*).

On the basis of this framework, we performed the fitting in multiple steps:

1) First step: In the first step, several models were used to fit the eCD4Ig and ADA data from uninfected animals in Monolix 2019R2 (see table S2). For initial values, we use *A_B_*(0) = 0, *A_T_*(0) = 0, and $A_{D}(0)=\frac{\lambda_{d}}{c_{d}}$. Because λ*_d_* and *c_d_* cannot be estimated together, we fixed *c_d_* = 0.0495/day (*15*). In addition, one-compartment and two-compartment models were also evaluated for completeness but did not describe the data well and were therefore not used. To further identify any covariates or correlations that influenced the model structure and were predictive of the PK dynamics, we used the default alternative model exploration in Monolix for the best model. When applicable, we assumed that *f_b_* = 60 *W* as the volume of blood in milliliters calculated as 60 times the weight of the animal (*W*).

2) Second step: Because the ADA observations were not available for infected RMs, we next used the best model from uninfected animals with correlations between *k_bt_* and *c_b_*, and *k_bd_* and λ*_d_*, which was determined as the best model in step 1 to fit combined eCD4Ig and ADA data from uninfected and infected animals. As the ADA data were not available for infected monkeys, we assumed that the parameter distribution of the parameters associated with ADA dynamics remained unaffected by fitting observations from infected and uninfected animals together. For that reason, we fixed them as estimated from fitting of the eCD4Ig and ADA data in uninfected animals in step 1. The population level fixed effect of remaining parameters for uninfected animals is also known from step 1, and therefore, we also fixed them. We then added covariates on kinetics parameters based on infected status (uninfected versus infected) and used the backward selection method to identify which parameters were significantly modified by the given covariate on infection status.

3) Third step: We next used the most parsimonious PK model and fixed parameters associated with the PK model from step 2. Next, we estimated parameters associated with the viral dynamics and identified the effect of eCD4Ig on the viral dynamics. During this estimation procedure, we fixed *d_I_* = 0.4/day (*48*–*50*), *c* = 23/day (*51*), *p* = 50,000 virions/cell per day (*52*), ϵ*ART* = 0.99 (*42*, *43*), $T(0)=\frac{\lambda_{T}}{d_{T}}$, *V*(0) = 0.05 × 10^1.16^ copies/ml, and $E(0)=\frac{\lambda_{E}}{d_{E}}$. In our model, *E* are a combination of short-lived and long-lived effector cells, and therefore, instead of fixing *d_E_* [to 1/day (*23*, *53*, *54*)], we fixed it to 0.003/day (*21*). This step was also necessary to allow for the presence of effector cells at the time of ATI given the viral dynamics differences between primary infection and after ATI.

4) Fourth step: We finally determined the parameters in the best PK model that can recapitulate eCD4Ig and ADA after eCD4Ig administration in the form of AAV vectors (model MA1 in table S10). During the estimation procedure, we fixed *c_d_* = 0.0495/day and *c_b_* = 0.37/day from step 1. We also fixed $d_{\text{AAV}}=24\times \frac{0.69}{2}$/day (*55*), λ*_N_* = 0.0001 cells/day (assumed), $c_{N}=\frac{0.69}{14}$/day (*56*, *57*), and δ*AAV* = 0.0003/day (*58*).

## Notes

View/request a protocol for this paper from *Bio-protocol*.
