# Supplementary material for: Estimation of the in vivo neutralization potency of eCD4Ig and conditions for AAV-mediated production for SHIV long-term remission
Source: Sci Adv. 2022 Jan 12;8(2):eabj5666. doi: 10.1126/sciadv.abj5666 (PMC8754410; doi:10.1126/sciadv.abj5666)
Supplement: Supplementary file 1 — Supplementary Materials and Methods Figs. S1 to S8 Tables S1 to S11 [file sciadv.abj5666_sm.pdf]

Science Advances
AAAS

Supplementary Materials for

Estimation of the in vivo neutralization potency of eCD4Ig and conditions for AAV-mediated production for SHIV long-term remission

Ashish Goyal, Matthew Gardner, Bryan T. Mayer, Keith R. Jerome, Michael Farzan, Joshua T. Schiffer, E. Fabian Cardozo-Ojeda*

*Corresponding author. Email: ecojeda@fredhutch.org

Published 12 January 2022, *Sci. Adv.* **8**, eabj5666 (2022)
DOI: 10.1126/sciadv.abj5666

The PDF file includes:

Supplementary Materials and Methods
Figs. S1 to S8
Tables S1 to S11

Other Supplementary Material for this manuscript includes the following:

Data files S1 to S4

Materials and Methods

Notes on the exploration of the model describing eCD4Ig PK dynamics

A total of 23 PK models were constructed to reproduce the concentration-time data for eCD4Ig and ADA in uninfected animals (description of all these models is provided in Table S1). The best model comprised of three compartments (eCD4Ig in blood, eCD4Ig in tissue and ADA in blood) was supported over one-, two- and four- compartment models by the data. As the data of uninfected animals involved multiple dose and variants of eCD4Ig, covariates and correlations were systematically searched in the best model. The data did not support any covariates suggesting that defined that the dynamics of eCD4Ig is independent of eCD4Ig variant and dose (**Figs S1 and S2**), and we can assume that all parameters follow the same population distribution independent of eCD4Ig variant and dose. For example, the addition of covariate on c_b on both dose and variants increased AIC from -203.4 to -58.1. Similarly, an addition of covariate on c_b on just dose increased AIC to -137.2 while the addition of covariate on c_b on just variants increased AIC to -61.8. The addition of covariate on k_{bd} on just variants increased AIC to -85.7. The addition of covariate on both k_{bd} and τ on just variants increased AIC to -47.5. The addition of covariate on both k_{tb} on both dose and variants increased AIC to -163.7. The addition of covariate on both k_{tb} on just variants increased AIC to -27.7. The addition of covariate on both k_{bt} on both dose and variants increased AIC to -156.5. However, strong correlations were identified between (k_{bt} and c_b) and (k_{bd} and λ_d) (r=0.65 and -0.94, Spearman Correlation) (**Table S1** and **Fig S3**).

Supplementary Figures

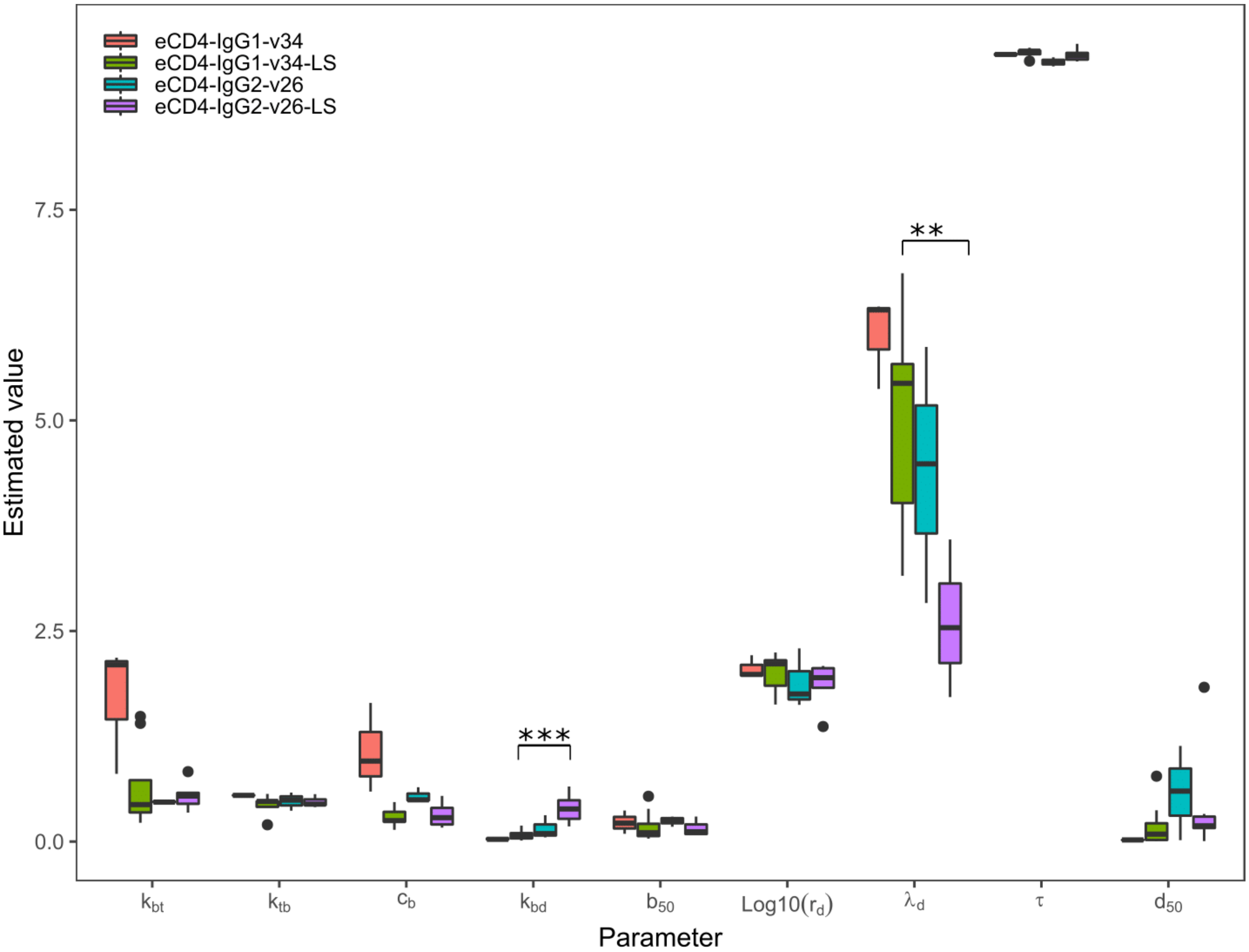

Figure S1: Boxplot for each parameter characterized by different variants (in uninfected animals). Wilcoxon rank sum test (with Bonferroni correction) to determine if median is significantly different between two groups (* implying p<0.05, ** implying p<0.01, *** implying p<0.001). The eCD4Ig is distributed from blood to tissue and back at rates k_{bt} and k_{tb}, respectively, and is cleared from the blood in the absence of ADA at rate c_b. Plasma levels of anti-eCD4Ig-drug antibodies (ADA) have a basal production with rate λ_d and a clearance rate of c_d. ADA expands with maximum rate r after a delay τ and saturates depending upon eCD4Ig levels governed by the parameter d_{50}. The presence of ADA mediates the clearance of eCD4Ig with maximum rate k_{bd} that is also saturated dependent on the eCD4Ig concentration based on parameter b_{50}. Only clearance of eCD4Ig mediated by ADA k_{bd} and the ADA basal production λ_d were different, but only between variants IgG1-v34-LS and IgG2 v26-LS.

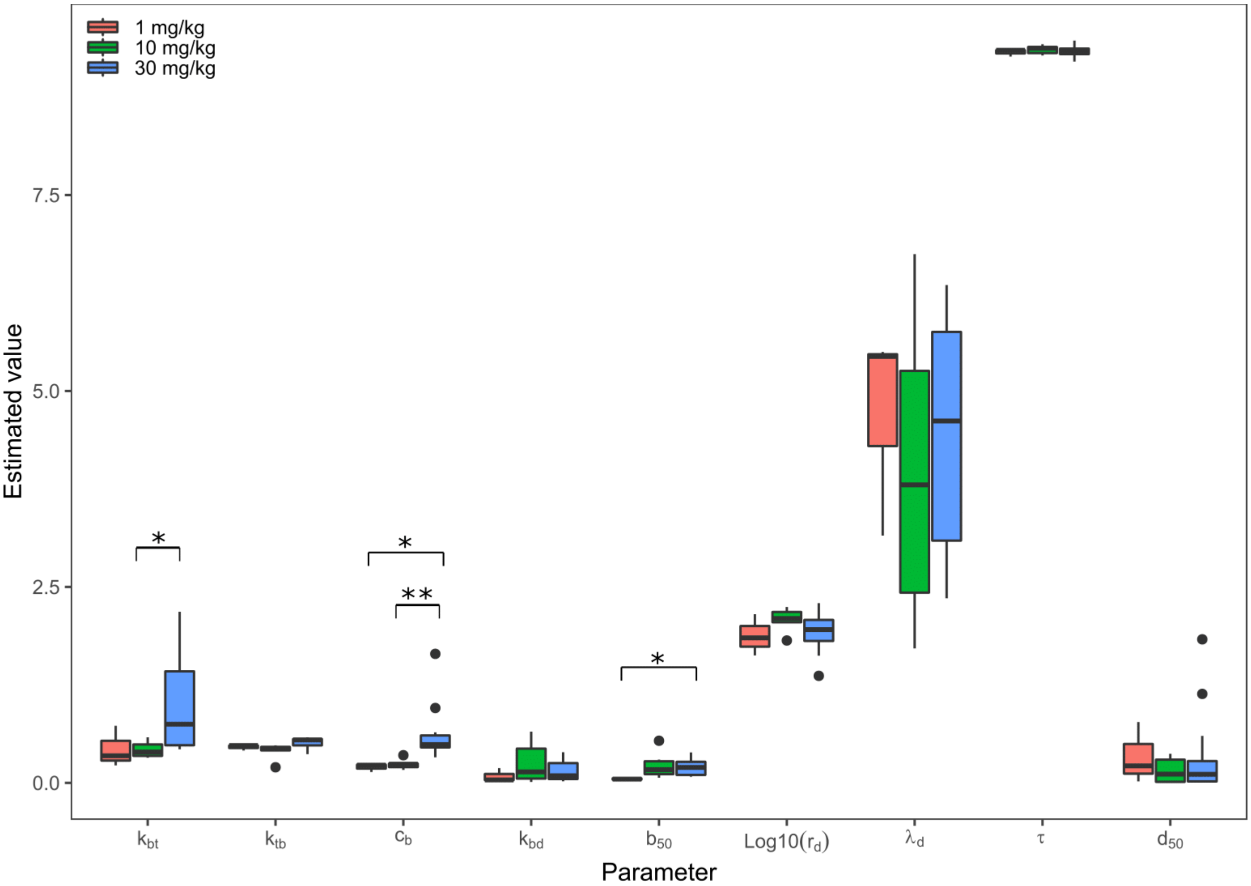

Figure S2: Boxplot for each parameter characterized by different doses (in uninfected animals). Wilcoxon rank sum test (with Bonferroni correction) to determine if median is significantly different between two groups (* implying p<0.05, ** implying p<0.01, *** implying p<0.001). The eCD4Ig is distributed from blood to tissue and back at rates k_{bt} and k_{tb}, respectively, and is cleared from the blood in the absence of ADA at rate c_b. Plasma levels of anti-eCD4Ig-drug antibodies (ADA) have a basal production with rate λ_d and a clearance rate of c_d. ADA expands with maximum rate r after a delay τ and saturates depending upon eCD4Ig levels governed by the parameter d_{50}. The presence of ADA mediates the clearance of eCD4Ig with maximum rate k_{bd} that is also saturated dependent on the eCD4Ig concentration based on parameter b_{50}. Only clearance of eCD4Ig in the absence of ADA c_b was significantly higher for a dose of 30 mg/kg.

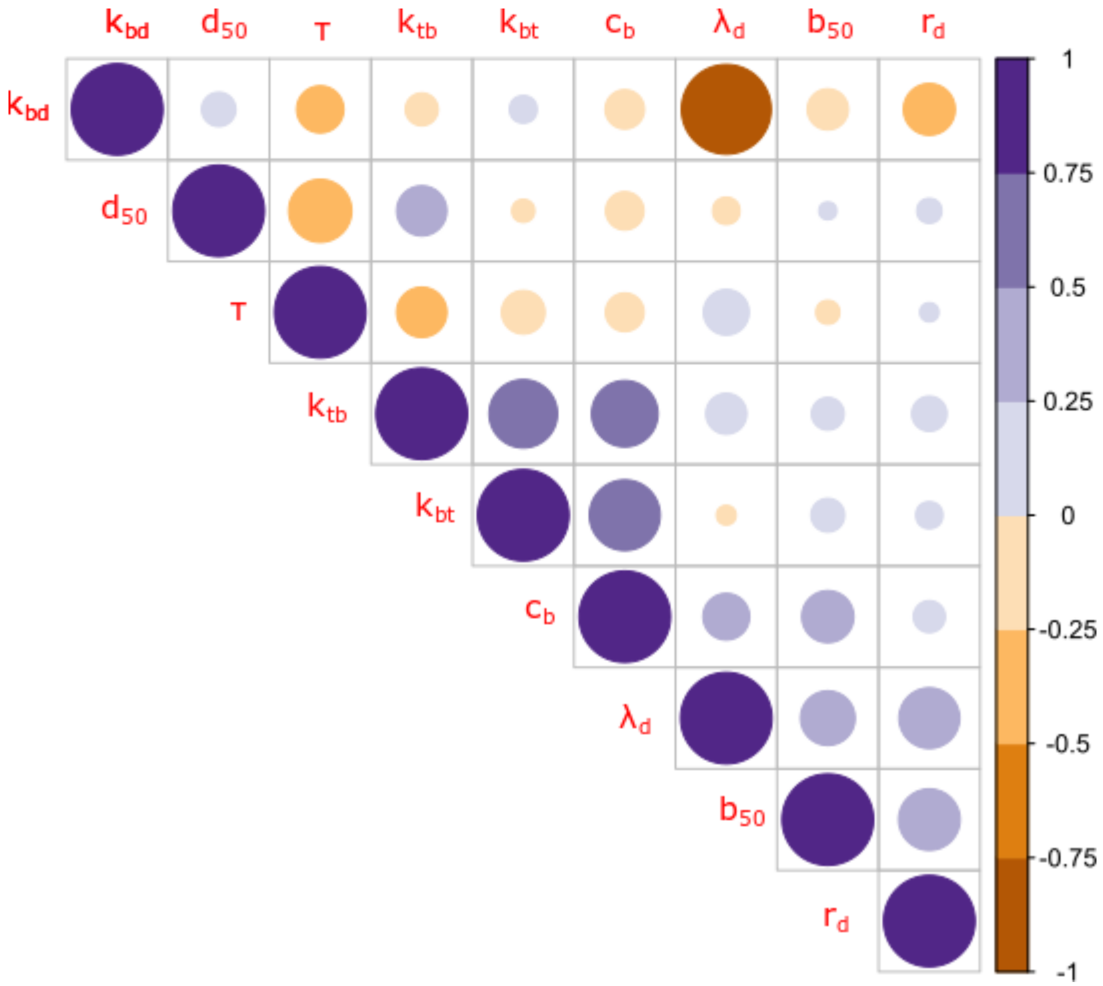

Figure S3: Spearman correlation between estimated parameters from the fitting of eCD4Ig and ADA in 21 uninfected animals.

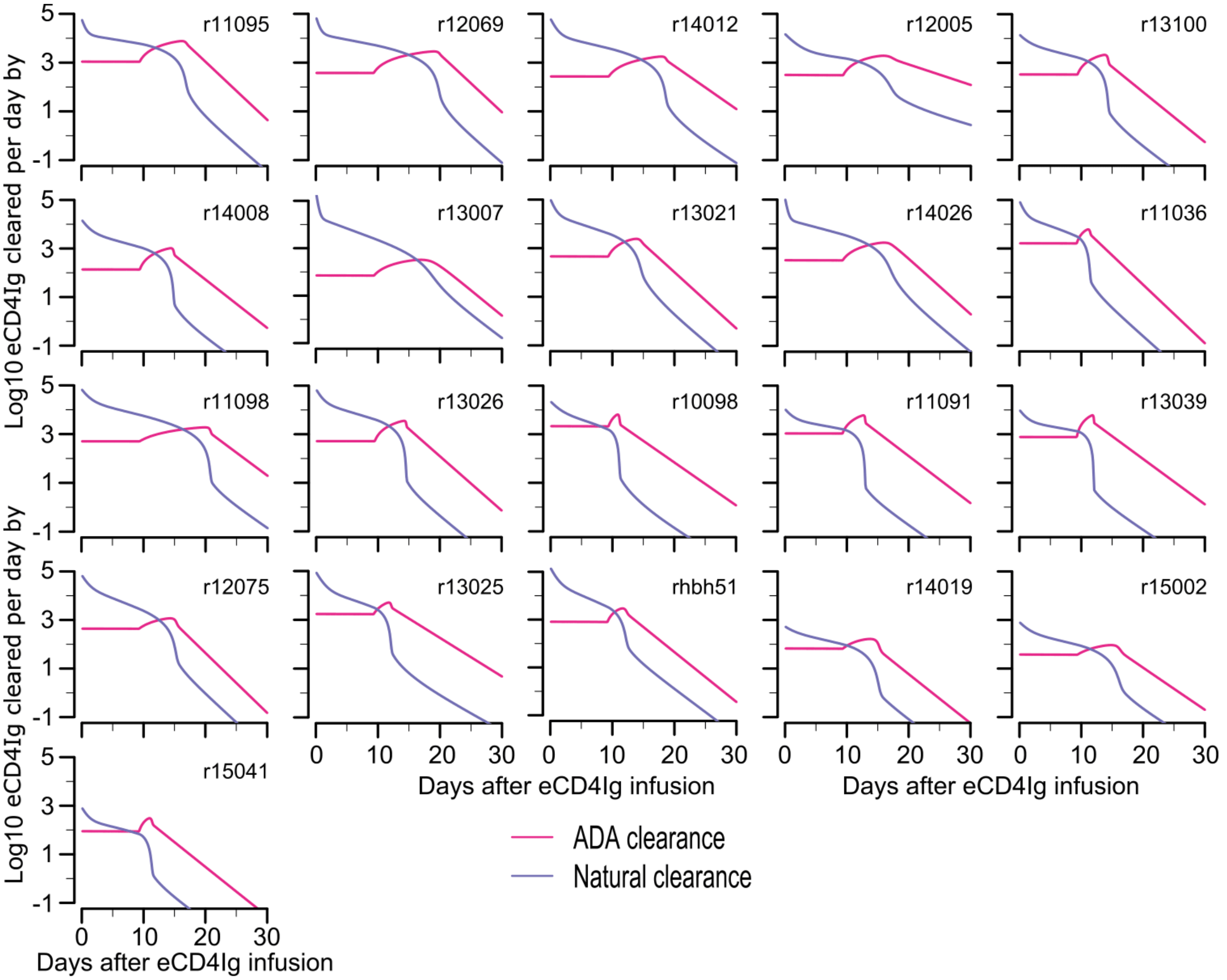

Figure S4: The amount of eCD4Ig (μg) cleared per day, (i) naturally (blue line), and (ii) ADA-mediated (pink line) for 21 uninfected animals. The concentration of eCD4Ig cleared per day follows the same trend as the concentration is derived by dividing the amount by a constant $f_b = 60W$ as the volume of blood in mL, where W is the weight of the animal.

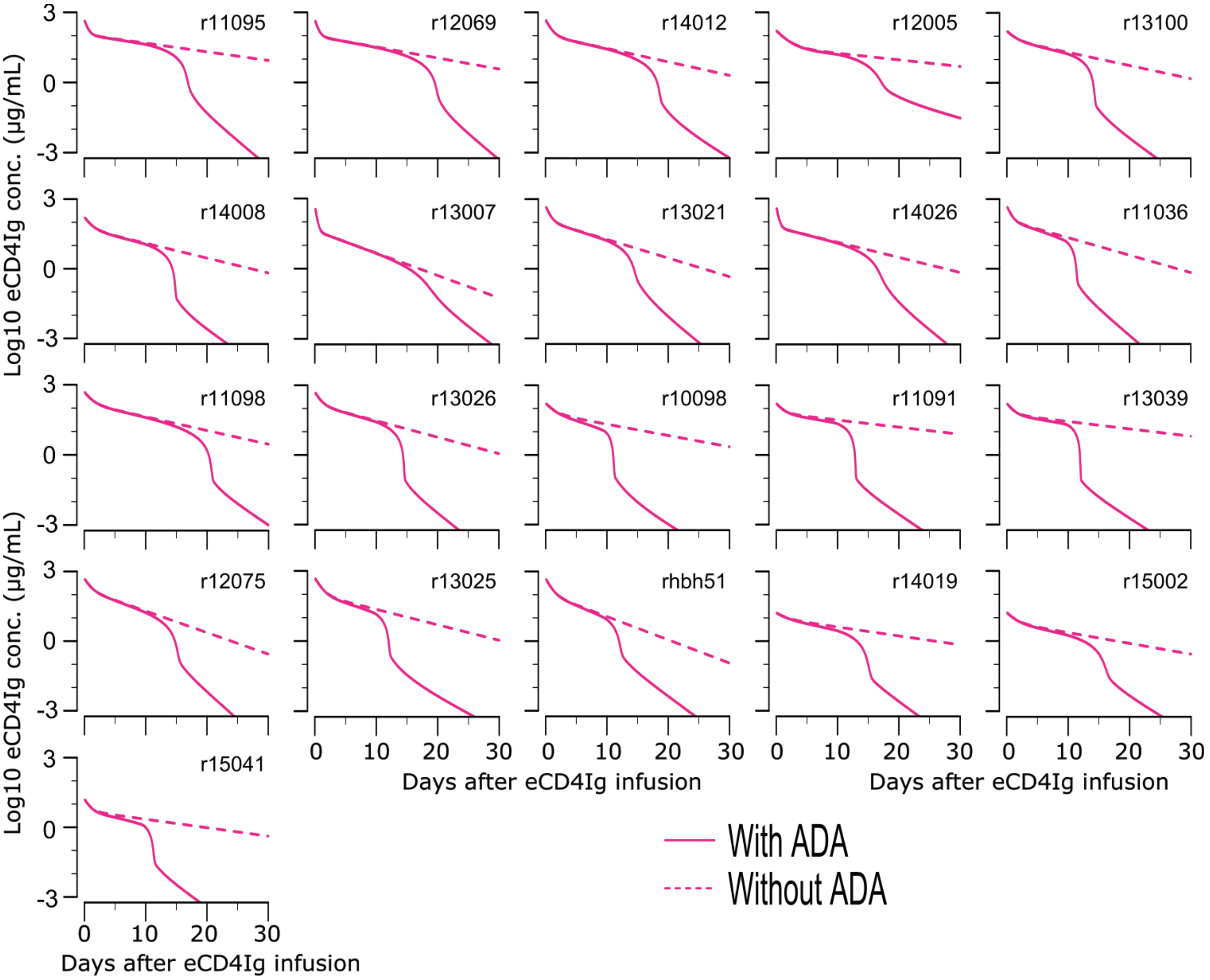

Figure S5: The impact of anti-drug antibodies (ADA) on the clearance of eCD4Ig in 21 uninfected animals. We employed the PK model that best recapitulates the observed data (model M9 in Table S1). Solid and dashed lines represent the dynamics of eCD4Ig in the presence (k_{bd} as estimated) and absence ($k_{bd} = 0$) of anti-drug antibodies, respectively.

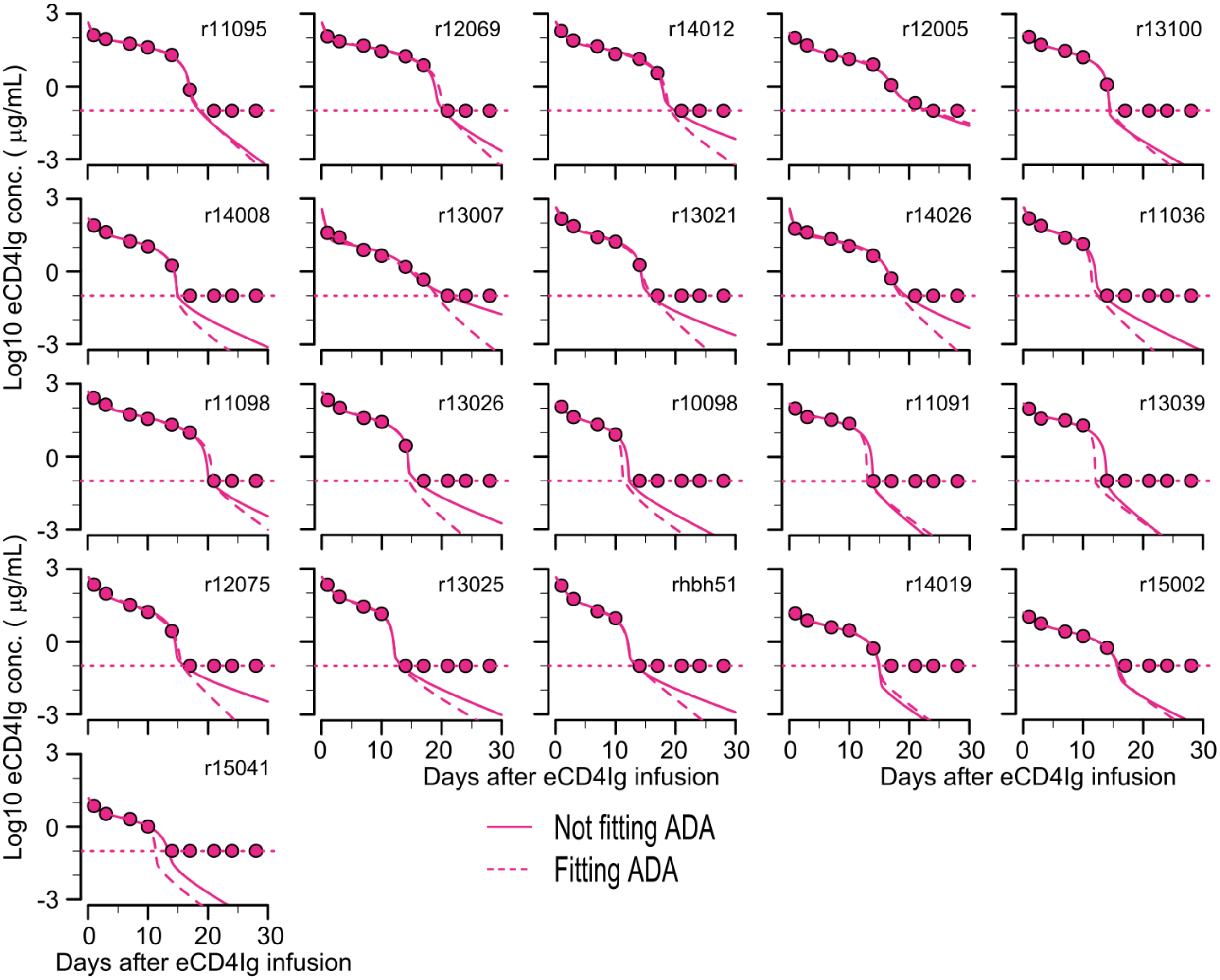

Figure S6: Fits to eCD4Ig data (round red markers) when we ignore ADA data in the fitting procedure (solid line, using model M3 in Table S1 without ADA equation) and when we include ADA data in the fitting procedure (dashed line, using model m9 in Table S1).

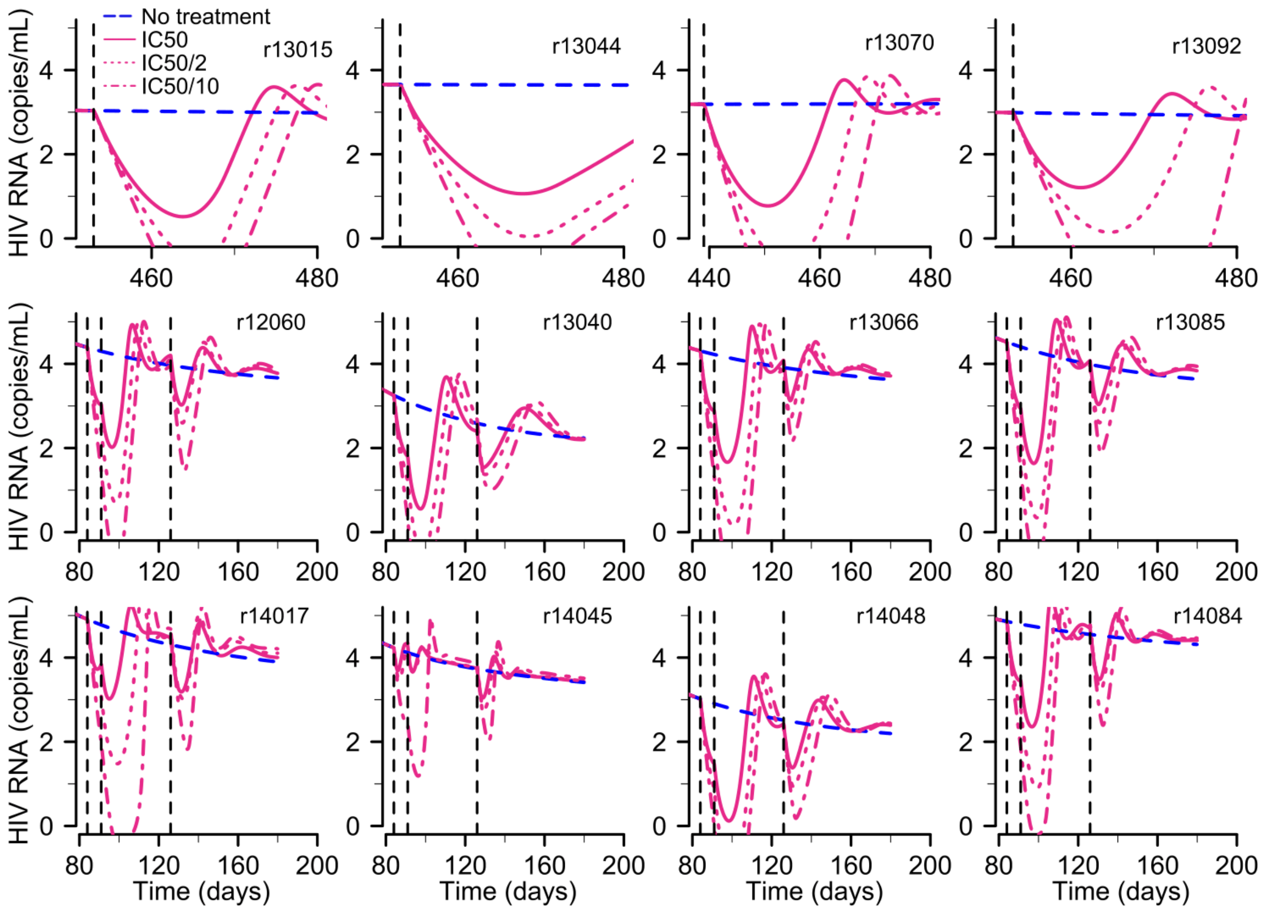

Figure S7: The viral load dynamics assuming no treatment (blue line), estimated EC50 (dashed pink line), assumed EC50 as half of the estimated EC50 (dashed pink line) and assumed EC50 ten times smaller than estimated EC50 (dashed dotted pink line).

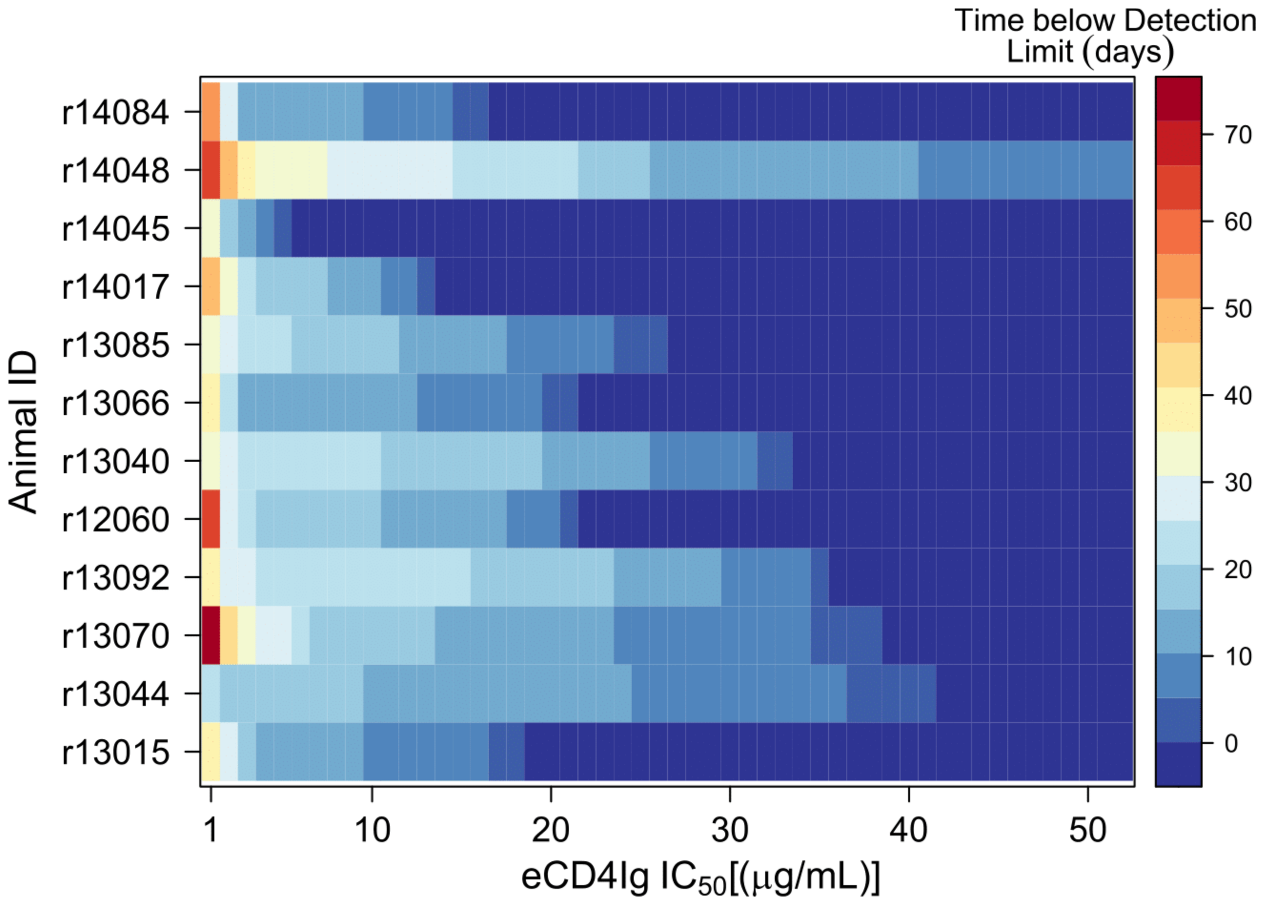

Figure S8: The duration for which virus stays below detection limit (in days) in relation to variation in vivo IC_{50} of eCD4Ig.

Supplementary Tables

Table S1: Characteristics of rhesus macaques in this study.

**Group Number (# of animals)**	**Infection Status**	**eCD4Ig administered (# of animals; IDs)**	**Different doses (# of animals; IDs)**
1 (n=21)	Uninfected	1. eCD4IgG1-v34-LS (**n=9**; r11095, r12069, r14012, r12005, r13100, r14008, r14019, r15002, r15041),<br><br>2. eCD4IgG1-v34 (**n=3**; r13007, r13021, r14026),<br><br>3. eCD4IgG2-v26-LS (**n=6**; r11036, r11098, r13026, r10098, r11091, r13039),<br><br>4. eCD4IgG2-v26 (**n=3**; r12075, r13025, rhbh51)	1. 30 mg/kg (**n=12**; r11095, r12069, r14012, r13007, r13021, r14026, r11036, r11098, r13026, r12075, r13025, rhbh51),<br><br>2. 10 mg/kg (**n=6**; r12005, r13100, r14008, r10098, r11091, r13039),<br><br>3. 1 mg/kg (**n=3**; r14019, r15002, r15041)
2 (n=12)	Infected with SHIV-AD8	1. eCD4Ig1-v34-LS (**n=2**;),<br><br>2. eCD4Ig1-v26-LS (**n=2**;)	1. 30 mg/kg (n=12)

Table S2: List of models explored to reproduce the dynamics of eCD4Ig in the blood (A_b) and anti-drug antibodies in the blood (A_d) in 21 uninfected rhesus macaques. These 21 uninfected were administered either different dose of the same variant of eCD4Ig antibodies or the same dose of the different variant of eCD4Ig but for the purpose of model exploration here, we assumed even different doses and different variants of eCD4Ig follow the same distribution. In these models, A_b and A_t denote the amount of eCD4Ig in blood and tissue (or some other compartment), respectively. Similarly, A_d represent the amount of anti-drug antibodies (ADA) in the blood. Among these models, the movement of eCD4Ig between the blood and the tissue compartment is modelled through rates k_{bt} and k_{tb} whereas eCD4Ig are cleared from the blood at rate c_b. There is also a background production rate of ADA at rate λ_d and a clearance rate of c_d. Specific model related components and assumptions are described in comments section in this Table.

**Model**	**Comments**	**AIC (ΔAIC)**
Model M1<br>$\frac{dA_b}{dt} = -k_{bt}A_b + k_{tb}A_t - c_bA_b$<br>$\frac{dA_t}{dt} = k_{bt}A_b - k_{tb}A_t$	*Model M1* assumes<br>(i) ADA is not produced in response to eCD4Ig.<br>(ii) ADA does not mediate clearance of eCD4Ig.	489.5 (693)

$\frac{dA_d}{dt} = \lambda_d - c_d A_d$<br><br>_Number of unknown parameters_: 5	Fitting results:<br>(i) A_b dynamics is represented by a straight line with slope k_{bt}.<br>(ii) A_d does not increase from its baseline value and the dynamics is missed completely.	
Model M2<br>$\frac{dA_b}{dt} = -k_{bt}A_b + k_{tb}A_t - k_{bd}A_b A_d$<br>$\frac{dA_t}{dt} = k_{bt}A_b - k_{tb}A_t$<br>$\frac{dA_d}{dt} = \lambda_d - c_d A_d$<br><br>_Number of unknown parameters_: 6	_Model M2_ assumes<br>(i) ADA is not produced in response to eCD4Ig.<br>(ii) ADA mediates clearance of eCD4Ig via mass-interaction term.<br><br>Fitting results:<br>(i) A_b dynamics is represented by a straight line with slope k_{bt}.<br>(ii) A_d does not increase from its baseline value and the dynamics is missed completely.	497.7 (701)
Model M3<br>$\frac{dA_b}{dt} = -k_{bt}A_b + k_{tb}A_t - \frac{k_{bd}A_b}{\left(1 + \frac{A_b}{b_{50}f_b}\right)} - c_b A_b$<br>$\frac{dA_t}{dt} = k_{bt}A_b - k_{tb}A_t$<br>$\frac{dA_d}{dt} = \lambda_d - c_d A_d$ or, $A_d(t) = \frac{\lambda_d}{c_d}$<br><br>_Number of unknown parameters_: 7	_Model M3_ assumes<br>(i) ADA is not produced in response to eCD4Ig.<br>(ii) ADA mediates clearance of eCD4Ig, and this clearance rate gets saturated as eCD4Ig in the blood increase.<br><br>Fitting results:<br>(i) A_b dynamics is captured.<br>(ii) A_d does not increase from its baseline value and the dynamics is missed completely.<br><br>_This model demonstrates the importance of fitting ADA data right from the start as models that miss ADA dynamics could still fit eCD4Ig in the blood but with completely different values of estimated parameters. Thus, ignoring ADA from early fitting could have a big impact on model projections._	315.2 (518)
Model M4<br>$\frac{dA_b}{dt} = -k_{bt}A_b + k_{tb}A_t - \frac{k_{bd}A_b A_d}{\left(1 + \frac{A_d}{b_{50}f_b}\right)} - c_b A_b$	_Model M4_ assumes<br>(i) ADA is not produced in response to eCD4Ig.<br>(ii) ADA mediates clearance of eCD4Ig, and this clearance rate gets saturated from increasing ADA. This is similar to ADA	507.3 (810)

$\frac{dA_t}{dt} = k_{bt}A_b - k_{tb}A_t$<br>$\frac{dA_d}{dt} = \lambda_d - c_d A_d$<br><br>*Number of unknown parameters*: 7	demonstrating a negative feedback to the production of ADA depending upon the amount of ADA in the blood.<br><br>Fitting results: B<br>(i) A_b dynamics is represented by a straight line.<br>(ii) A_d does not increase from its baseline value and the dynamics is missed completely.	
Model M5<br>$\frac{dA_b}{dt} = -k_{bt}A_b + k_{tb}A_t - \frac{k_{bd}A_b A_d}{\left(1 + \frac{A_b}{b_{50}f_b}\right)} - c_b A_b$<br>$\frac{dA_t}{dt} = k_{bt}A_b - k_{tb}A_t$<br>$\frac{dA_d}{dt} = \lambda_d + rA_b - c_d A_d$<br><br>*Number of unknown parameters*: 8	*Model M5* assumes<br>(i) ADA is produced in response to eCD4Ig in a linear fashion.<br>(ii) ADA mediates clearance of eCD4Ig, and this clearance rate gets saturated as eCD4Ig in the blood increase.<br><br>Fitting results:<br>(i) A_b dynamics is captured.<br>(ii) A_d increases from its baseline value but the dynamics is still not fully captured.	328.2<br>(531)
Model M6<br>$\frac{dA_b}{dt} = -k_{bt}A_b + k_{tb}A_t - \frac{k_{bd}A_b A_d}{\left(1 + \frac{A_b}{b_{50}f_b}\right)} - c_b A_b$<br>$\frac{dA_t}{dt} = k_{bt}A_b - k_{tb}A_t$<br>$\frac{dA_d}{dt} = \lambda_d + rA_b A_d - c_d A_d$<br><br>*Number of unknown parameters*: 8	*Model M6* assumes<br>(i) ADA is produced in response to eCD4Ig but in a non-linear fashion.<br>(ii) ADA mediates clearance of eCD4Ig, and this clearance rate gets saturated as eCD4Ig in the blood increase.<br><br>Fitting results:<br>(i) A_b dynamics is captured.<br>(ii) A_d increases from its baseline value but the dynamics is still not fully captured.	314.5<br>(517)
Model M7<br>$\frac{dA_b}{dt} = -k_{bt}A_b + k_{tb}A_t - \frac{k_{bd}A_b A_d}{\left(1 + \frac{A_b}{b_{50}f_b}\right)} - c_b A_b$<br>$\frac{dA_t}{dt} = k_{bt}A_b - k_{tb}A_t$<br>$\frac{dA_d}{dt} = \lambda_d + r(t)A_b - c_d A_d$<br>where,<br>$r(t) = 0$ is $t < \tau$ but $r(t) = r_d$ if $t \geq \tau$<br><br>*Number of unknown parameters*: 9	*Model M7* assumes<br>(i) ADA is produced in response to eCD4Ig in a linear fashion and there is an **on-off switch** representing that it takes some time for B cells to recognize antibodies, process them and start producing ADA in response.<br>(ii) ADA mediates clearance of eCD4Ig, and this clearance rate gets saturated as eCD4Ig in the blood increase.<br><br>Fitting results:<br>(i) A_b dynamics is captured.	-134.1<br>(69.3)

	(ii) A_d fits improve but a sudden increase from its baseline value does not match the early growth of A_d.	
Model M8<br>$\frac{dA_b}{dt} = -k_{bt}A_b + k_{tb}A_t - \frac{k_{bd}A_bA_d}{\left(1+\frac{A_b}{b_{50}f_b}\right)} - c_bA_b$<br>$\frac{dA_t}{dt} = k_{bt}A_b - k_{tb}A_t$<br>$\frac{dA_d}{dt} = \lambda_d + r(t)A_bA_d - c_dA_d$<br>where,<br>$r(t) = 0$ is $t < \tau$ but $r(t) = r_d$ if $t \geq \tau$<br><br>*Number of unknown parameters*: 9	*Model M8* assumes<br>(i) ADA is produced in response to eCD4Ig in a non-linear eCD4Igfashion and there is an **on-off switch** representing that it takes some time for memory B cells to recognize antibodies, process them and start producing ADA in response.<br>(ii) ADA mediates clearance of eCD4Ig, and this clearance rate gets saturated as eCD4Ig in the blood increase.<br><br>Fitting results:<br>(i) A_b dynamics is captured but not as well as in the case of A_d production in a linear fashion to A_b with an on-off switch.<br>(ii) A_d fits improve but a sudden increase from its baseline value does not match the early growth of A_d.	34.9<br>(238)
Model M9<br>$\frac{dA_b}{dt} = -k_{bt}A_b + k_{tb}A_t - \frac{k_{bd}A_bA_d}{\left(1+\frac{A_b}{b_{50}f_b}\right)} - c_bA_b$<br>$\frac{dA_t}{dt} = k_{bt}A_b - k_{tb}A_t$<br>$\frac{dA_d}{dt} = \lambda_d + \frac{r(t)A_b}{(d_{50}f_b + A_b)} - c_dA_d$<br>where,<br>$r(t) = 0$ is $t < \tau$ but $r(t) = r_d$ if $t \geq \tau$<br><br>*Number of unknown parameters*: 10	*Model M9* assumes<br>(i) ADA is produced in response to eCD4Ig in a linear fashion and this production rate has a negative feedback regulated by the amount of eCD4Ig. This term allows immune system to restrict from having a large number of ADA even when eCD4Ig keep increasing. There is also an **on-off switch** that controls the production of ADA in response to eCD4Ig.<br>(ii) ADA mediates clearance of eCD4Ig antibodies, and this clearance rate gets saturated as eCD4Ig in the blood increase.<br><br>Fitting results:<br>(i) A_b dynamics is captured.<br>(ii) A_d dynamics is captured.<br>(iii) *Best model fits.*	-203.4<br>(0)
Model M10<br>$\frac{dA_b}{dt} = -k_{bt}A_b + k_{tb}A_t - c_bA_b$<br>$\frac{dA_t}{dt} = k_{bt}A_b - k_{tb}A_t$	*Model M10* assumes<br>(i) ADA is produced in response to eCD4Ig in a linear fashion and there is also an **on-off switch** that controls the production of ADA in response to eCD4Ig.	113.3<br>(317)

$\frac{dA_d}{dt} = \lambda_d + r(t)A_b - c_d A_d$<br>where,<br>$r(t) = 0$ is $t < \tau$ but $r(t) = r_d$ if $t \geq \tau$<br><br>*Number of unknown parameters*: 7	(ii) ADA does not mediate clearance of eCD4Ig.<br><br>Fitting results:<br>(i) A_b dynamics is represented by a straight line with slope k_{bt}.<br>(ii) A_d fits are decent but a sudden increase from its baseline value does not match the early growth of A_d.	
Model M11<br>$\frac{dA_b}{dt} = -k_{bt}A_b + k_{tb}A_t - k_{bd}A_bA_d - c_bA_b$<br>$\frac{dA_t}{dt} = k_{bt}A_b - k_{tb}A_t$<br>$\frac{dA_d}{dt} = \lambda_d + r(t)A_b - c_d A_d$<br>where,<br>$r(t) = 0$ is $t < \tau$ but $r(t) = r_d$ if $t \geq \tau$<br><br>*Number of unknown parameters*: 8	*Model M11* assumes<br>(i) ADA is produced in response to eCD4Ig in a linear fashion and there is also an **on-off switch** that controls the production of ADA in response to eCD4Ig.<br>(ii) ADA mediates clearance of eCD4Ig via mass-interaction term.<br><br>Fitting results:<br>(i) A_b dynamics is represented by a straight line with slope k_{bt}.<br>(ii) A_d fits are decent but a sudden increase from its baseline value does not match the early growth of A_d.	-38.7 (164)
Model M12<br>$\frac{dA_b}{dt} = -k_{bt}A_b + k_{tb}A_t - c_bA_b$<br>$\frac{dA_t}{dt} = k_{bt}A_b - k_{tb}A_t$<br>$\frac{dA_d}{dt} = \lambda_d + \frac{r(t)A_b}{(d_{50}f_b + A_b)} - c_d A_d$<br>where,<br>$r(t) = 0$ is $t < \tau$ but $r(t) = r_d$ if $t \geq \tau$<br><br>*Number of unknown parameters*: 8	*Model M12* assumes<br>(i) ADA is produced in response to eCD4Ig in a linear fashion and this production rate has a negative feedback regulated by the amount of eCD4Ig. This term allows immune system to restrict from having a large number of ADA even when eCD4Ig keep increasing. There is also an **on-off switch** that controls the production of ADA in response to eCD4Ig.<br>(ii) ADA does not mediate clearance of eCD4Ig.<br><br>Fitting results:<br>(i) A_b dynamics is represented by a straight line with slope k_{bt}.<br>(ii) A_d fits are decent but not as well as a model M9.	54.6 (258)
Model M13	*Model M13* assumes<br>(i) ADA is produced in response to eCD4Ig in a linear fashion and this	-85.3 (118)

$\frac{dA_b}{dt} = -k_{bt}A_b + k_{tb}A_t - k_{bd}A_bA_d - c_bA_b$<br>$\frac{dA_t}{dt} = k_{bt}A_b - k_{tb}A_t$<br>$\frac{dA_d}{dt} = \lambda_d + \frac{r(t)A_b}{(d_{50}f_b + A_b)} - c_dA_d$<br>where,<br>$r(t) = 0$ is $t < \tau$ but $r(t) = r_d$ if $t \geq \tau$<br><br>*Number of unknown parameters*: 9	production rate has a negative feedback regulated by the amount of eCD4Ig. This term allows immune system to restrict from having a large number of ADA even when eCD4Ig keep increasing. There is also an **on-off switch** that controls the production of ADA in response to eCD4Ig.<br>(ii) ADA mediates clearance of eCD4Ig via mass-interaction term.<br><br>Fitting results:<br>(i) A_b dynamics is captured but not as well as a model M9.<br>(ii) A_d fits are decent but not as well as a model M9.	
Model M14<br>$\frac{dA_b}{dt} = -k_{bt}A_b + k_{tb}A_t - \frac{k_{bd}A_bA_d}{\left(1 + \frac{A_b}{b_{50}f_b}\right)} - c_bA_b$<br>$\frac{dA_t}{dt} = k_{bt}A_b - k_{tb}A_t$<br>$\frac{dA_d}{dt} = \lambda_d + rA_b(t - \tau) - c_dA_d$<br><br>*Number of unknown parameters*: 9	*Model M14* assumes<br>(i) ADA is produced in response to eCD4Ig in a linear fashion and this production rate has a **delay of τ** recognizing time delay between eCD4Ig presentation to B cells and ADA production.<br>(ii) ADA mediates clearance of eCD4Ig, and this clearance rate gets saturated as eCD4Ig in the blood increase.<br><br>Fitting results:<br>(i) A_b dynamics is captured but not as well as a model M9.<br>(ii) A_d fits are decent but a sudden increase from its baseline value does not match the early growth of A_d.	-85.6 (118)
Model M15<br>$\frac{dA_b}{dt} = -k_{bt}A_b + k_{tb}A_t - \frac{k_{bd}A_bA_d}{\left(1 + \frac{A_d}{b_{50}f_b}\right)} - c_bA_b$<br>$\frac{dA_t}{dt} = k_{bt}A_b - k_{tb}A_t$<br>$\frac{dA_d}{dt} = \lambda_d + r(t)A_b - c_dA_d$<br>where,<br>$r(t) = 0$ is $t < \tau$ but $r(t) = r_d$ if $t \geq \tau$<br><br>*Number of unknown parameters*: 9	*Model M15* assumes<br>(i) ADA is produced in response to eCD4Ig in a linear fashion and there is also an **on-off switch** that controls the production of ADA in response to eCD4Ig.<br>(ii) ADA mediates clearance of eCD4Ig, and this clearance rate gets saturated as eCD4Ig in the blood increase.<br><br>Fitting results:<br>(i) A_b dynamics is captured but not as well as a model M9.	-44.3 (159)

	(ii) A_d fits are decent but a sudden increase from its baseline value does not match the early growth of A_d.	
Model M16<br>$\frac{dA_b}{dt} = -k_{bt}A_b + k_{tb}A_t - \frac{k_{bd}A_bA_d}{\left(1+\frac{A_b}{b_{50}f_b}\right)} - c_bA_b$<br>$\frac{dA_t}{dt} = k_{bt}A_b - k_{tb}A_t$<br>$\frac{dA_d}{dt} = \lambda_d + r(t)A_bA_d - c_dA_d$<br>where,<br>$r(t) = 0$ is $t < \tau$ but $r(t) = r_d$ if $t \geq \tau$<br><br>*Number of unknown parameters*: 9	*Model M16* assumes<br>(i) ADA is produced in response to eCD4Ig in a non-linear fashion and there is also an **on-off switch** that controls the production of ADA in response to eCD4Ig.<br>(ii) ADA mediates clearance of eCD4Ig, and this clearance rate gets saturated as eCD4Ig in the blood increase.<br><br>Fitting results:<br>(i) A_b dynamics is captured.<br>(ii) A_d fits are decent but a sudden increase from its baseline value does not match the early growth of A_d.	-130.0 (73)
Model M17<br>$\frac{dA_b}{dt} = -k_{bt}A_b + k_{tb}A_t - \frac{k_{bd}A_b^nA_d}{\left(1+\left(\frac{A_b}{b_{50}f_b}\right)^n\right)} - c_bA_b$<br>$\frac{dA_t}{dt} = k_{bt}A_b - k_{tb}A_t$<br>$\frac{dA_d}{dt} = \lambda_d + r(t)A_b - c_dA_d$<br>where,<br>$r(t) = 0$ is $t < \tau$ but $r(t) = r_d$ if $t \geq \tau$<br><br>*Number of unknown parameters*: 10	*Model M17* assumes<br>(i) ADA is produced in response to eCD4Ig in a linear fashion and there is also an **on-off switch** that controls the production of ADA in response to eCD4Ig.<br>(ii) ADA mediates clearance of eCD4Ig, and this clearance rate gets saturated as eCD4Ig in the blood increase. We also assumed here that once an ADA gets attached to an eCD4Ig antibody, it can alter the binding capacity of another ADA to the same eCD4Ig antibody, represented by the hill coefficient 'n'.<br><br>Fitting results:<br>(i) A_b dynamics is captured but not as well as a model M9.<br>(ii) A_d fits are decent but a sudden increase from its baseline value does not match the early growth of A_d.	-38.2 (165)
Model M18<br>$\frac{dA_b}{dt} = -k_{bt}A_b + k_{tb}A_t - \frac{k_{bd}A_b^nA_d}{\left(1+\left(\frac{A_b}{b_{50}f_b}\right)^n\right)} - c_bA_b$	*Model M18* assumes<br>(i) ADA is produced in response to eCD4Ig in a non-linear fashion and there is also an **on-off switch** that controls the production of ADA in response to eCD4Ig.	-33.5 (170)

$\frac{dA_t}{dt} = k_{bt}A_b - k_{tb}A_t$<br>$\frac{dA_d}{dt} = \lambda_d + r(t)A_bA_d - c_dA_d$<br>where,<br>$r(t) = 0$ is $t < \tau$ but $r(t) = r_d$ if $t \geq \tau$<br><br>_Number of unknown parameters_: 10	(ii) ADA mediates clearance of eCD4Ig, and this clearance rate gets saturated as eCD4Ig in the blood increase. We also assumed here that once an ADA gets attached to an eCD4Ig antibody, it can alter the binding capacity of another ADA to the same eCD4Ig antibody, represented by the hill coefficient 'n'.<br><br>Fitting results:<br>(i) A_b dynamics is captured but not as well as a model M9.<br>(ii) A_d fits are decent but a sudden increase from its baseline value does not match the early growth of A_d.	
Model M19<br>$\frac{dA_b}{dt} = -k_{bt}A_b + k_{tb}A_t - \frac{k_{bd}A_bA_d}{\left(1 + \frac{A_d}{b_{50}f_b}\right)} - c_bA_b$<br>$\frac{dA_t}{dt} = k_{bt}A_b - k_{tb}A_t$<br>$\frac{dA_d}{dt} = \lambda_d + \frac{r(t)A_b}{(d_{50}f_b + A_b)} - c_dA_d$<br>where,<br>$r(t) = 0$ is $t < \tau$ but $r(t) = r_d$ if $t \geq \tau$<br><br>_Number of unknown parameters_: 10	_Model M19 (alternative to Model M9)_ assumes<br>(i) ADA is produced in response to eCD4Ig in a non-linear fashion and there is also an **on-off switch** that controls the production of ADA in response to eCD4Ig.<br>(ii) ADA mediates clearance of eCD4Ig, and this clearance rate gets saturated as the amount of ADA in the blood increase.<br><br>Fitting results:<br>(i) A_b dynamics is captured but not as well as a model M9.<br>(ii) A_d fits are decent but not as well as a model M9.	-86.0 (117)
Model M20<br>$\frac{dA_b}{dt} = -k_{bt}A_b + k_{tb}A_t - \frac{k_{bd}A_b^nA_d}{\left(1 + \left(\frac{A_b}{b_{50}f_b}\right)^n\right)} - c_bA_b$<br>$\frac{dA_t}{dt} = k_{bt}A_b - k_{tb}A_t$<br>$\frac{dA_d}{dt} = \lambda_d + \frac{r(t)A_b}{(d_{50}f_b + A_b)} - c_dA_d$<br>where,<br>$r(t) = 0$ is $t < \tau$ but $r(t) = r_d$ if $t \geq \tau$<br><br>_Number of unknown parameters_: 11	_Model M20_ assumes<br>(i) ADA is produced in response to eCD4Ig in a linear fashion and this production rate has a negative feedback regulated by the amount of eCD4Ig. This term allows immune system to restrict from having a large number of ADA even when eCD4Ig keep increasing. There is also an **on-off switch** that controls the production of ADA in response to eCD4Ig.<br>(ii) ADA mediates clearance of eCD4Ig, and this clearance rate gets saturated as eCD4Ig in the blood increase. We also assumed here that once an ADA gets	-200.1 (3.3)

	attached to an eCD4Ig antibody, it can alter the binding capacity of another ADA to the same eCD4Ig antibody, represented by the hill coefficient 'n'.<br><br>Fitting results:<br>(i) A_b dynamics is captured.<br>(ii) A_d dynamics is captured.<br>(iii) Simulations yield $n \sim 1$, making M20 equivalent to M9 but with one more parameter.	
<u>*Model M21*</u><br>$\frac{dA_b}{dt} = -k_{bt}A_b + k_{tb}A_t - \frac{k_{bd}A_bA_d}{\left(1 + \frac{A_b}{b_{50}f_b}\right)} - c_bA_b$<br>$\frac{dA_t}{dt} = k_{bt}A_b - k_{tb}A_t$<br>$\frac{dA_d}{dt} = \lambda_d + \frac{r(t)A_bA_d}{(d_{50}f_b + A_b)} - c_dA_d$<br>where,<br>$r(t) = 0$ is $t < \tau$ but $r(t) = r_d$ if $t \geq \tau$<br><br><u>*Number of unknown parameters*</u>: 10	<u>*Model M21*</u> assumes<br>(i) ADA is produced in response to eCD4Ig in a non-linear fashion and this production rate has a negative feedback regulated by the amount of eCD4Ig. This term allows immune system to restrict from having a large number of ADA even when eCD4Ig keep increasing. There is also an **on-off switch** that controls the production of ADA in response to eCD4Ig.<br>(ii) ADA mediates clearance of eCD4Ig, and this clearance rate gets saturated as eCD4Ig in the blood increase.<br><br>Fitting results:<br>(i) A_b dynamics is captured but only slightly inferior to model M9.<br>(ii) A_d dynamics is captured but only slightly inferior to model M9.	-196.9<br>(6.5)
<u>*Model M22*</u><br>$\frac{dA_b}{dt} = -k_{bt}A_b + k_{tb}A_t - \frac{k_{bd}A_bA_d}{\left(1 + \frac{A_b}{b_{50}f_b}\right)} - c_bA_b$<br>$\frac{dA_t}{dt} = k_{bt}A_b - k_{tb}A_t$<br>$\frac{dA_d}{dt} = \lambda_d + \frac{rA_b(t-\tau)}{(d_{50}f_b + A_b(t-\tau))} - c_dA_d$<br><br><u>*Number of unknown parameters*</u>: 10	<u>*Model M22*</u> assumes<br>(i) ADA is produced in response to eCD4Ig in a linear fashion and this production rate has a **delay of τ** recognizing time delay between eCD4Ig presentation to B cells and ADA production. This production rate also has a negative feedback regulated by the amount of eCD4Ig with the same delay 'τ'. This term allows immune system to restrict from having a large number of ADA even when eCD4Ig keep increasing.<br>(ii) ADA mediates clearance of eCD4Ig, and this clearance rate gets saturated as eCD4Ig in the blood increase.	-112.2<br>(91)

	Fitting results:<br>(i) A_b dynamics is captured but not good as the model M9.<br>(ii) A_d dynamics is captured but not good as the model M9.	
<u>*Model M23*</u><br>$$\frac{dA_b}{dt} = -k_{bt}A_b + k_{tb}A_t - \frac{k_{bd}A_b^n A_d}{\left(1 + \left(\frac{A_b}{b_{50}f_b}\right)^n\right)} - c_b A_b$$<br>$$\frac{dA_t}{dt} = k_{bt}A_b - k_{tb}A_t$$<br>$$\frac{dA_d}{dt} = \lambda_d + \frac{rA_b(t-\tau)}{(d_{50}f_b + A_b(t-\tau))} - c_d A_d$$<br><u>*Number of unknown parameters*</u>: 11	<u>*Model M23*</u> assumes<br>(i) ADA is produced in response to eCD4Ig in a linear fashion and this production rate has a **delay of τ** recognizing time delay between eCD4Ig presentation to B cells and ADA production. This production rate also has a negative feedback regulated by the amount of eCD4Ig with the same delay 'τ'. This term allows immune system to restrict from having a large number of ADA even when eCD4Ig keep increasing.<br>(ii) ADA mediates clearance of eCD4Ig, and this clearance rate gets saturated as eCD4Ig in the blood increase. We also assumed here that once an ADA gets attached to an eCD4Ig antibody, it can alter the binding capacity of another ADA to the same eCD4Ig antibody, represented by the hill coefficient 'n'.<br><br>Fitting results:<br>(i) A_b dynamics is captured but not good as the model M9.<br>(ii) A_d dynamics is captured but not good as the model M9.	-103.3 (100)

Table S3: The parameter values estimated individually for all uninfected animals using the PK model that best recapitulates the observed data (model M9 in Table S1). These parameters are estimated by fitting eCD4Ig and anti-drug antibodies data from 21 uninfected rhesus macaques in Monolix R2019R2. We also fixed $c_d = 0.0495$/day. Here, $t_{1/2}$ represents the terminal half-life in days.

ID	k_{bt}	k_{tb}	c_b	k_{bd}	b_{50}	r_d	λ_d	τ	d_{50}	Protein	Dose	$t_{1/2}$
r11095	1.4	0.6	0.3	0.06	0.39	133.1	5.7	9.4	0.13	eCD4IgG1-v34-LS	30 mg/kg	8.1
r12069	1.5	0.6	0.5	0.09	0.13	89.6	4.7	9.3	0.09	eCD4IgG1-v34-LS	30 mg/kg	6.4
r14012	0.7	0.4	0.5	0.12	0.10	67.6	3.9	9.4	0.00	eCD4IgG1-v34-LS	30 mg/kg	5.2
r12005	0.3	0.2	0.2	0.01	0.54	160.0	6.7	9.4	0.37	eCD4IgG1-v34-LS	10 mg/kg	10.6

r13100	0.3	0.5	0.2	0.04	0.21	175.4	5.7	9.4	0.02	eCD4IgG1-v34-LS	10 mg/kg	5.3
r14008	0.4	0.5	0.4	0.10	0.07	128.7	4.0	9.4	0.01	eCD4IgG1-v34-LS	10 mg/kg	4.7
r13007	2.1	0.5	1.6	0.02	0.09	91.1	6.3	9.4	0.02	eCD4IgG1-v34	30 mg/kg	3.1
r13021	0.8	0.5	0.6	0.03	0.37	162.7	6.4	9.3	0.02	eCD4IgG1-v34	30 mg/kg	3.8
r14026	2.2	0.6	1.0	0.05	0.22	96.3	5.4	9.3	0.02	eCD4IgG1-v34	30 mg/kg	4.6
r11036	0.8	0.6	0.5	0.38	0.23	115.9	2.7	9.3	1.83	eCD4IgG2-v26-LS	30 mg/kg	4.0
r11098	0.4	0.4	0.3	0.23	0.08	23.2	3.2	9.4	0.16	eCD4IgG2-v26-LS	30 mg/kg	5.1
r13026	0.6	0.5	0.4	0.39	0.08	71.2	2.4	9.5	0.17	eCD4IgG2-v26-LS	30 mg/kg	4.3
r10098	0.3	0.4	0.2	0.18	0.30	121.3	3.6	9.4	0.01	eCD4IgG2-v26-LS	10 mg/kg	6.2
r11091	0.5	0.4	0.2	0.52	0.13	65.5	2.0	9.3	0.20	eCD4IgG2-v26-LS	10 mg/kg	9.8
r13039	0.6	0.4	0.2	0.65	0.11	109.2	1.7	9.3	0.33	eCD4IgG2-v26-LS	10 mg/kg	9.5
r12075	0.5	0.6	0.5	0.09	0.18	41.9	4.5	9.3	0.60	eCD4IgG2-v26	30 mg/kg	3.2
r13025	0.5	0.4	0.5	0.31	0.26	56.5	2.8	9.3	0.02	eCD4IgG2-v26	30 mg/kg	4.6
rhbh51	0.5	0.5	0.6	0.05	0.30	196.2	5.9	9.2	1.14	eCD4IgG2-v26	30 mg/kg	3.0
r14019	0.2	0.5	0.1	0.04	0.07	70.8	5.5	9.3	0.78	eCD4IgG1-v34-LS	1 mg/kg	7.9
r15002	0.3	0.4	0.2	0.03	0.05	42.3	5.4	9.3	0.02	eCD4IgG1-v34-LS	1 mg/kg	6.5
r15041	0.7	0.5	0.2	0.19	0.03	141.9	3.2	9.3	0.22	eCD4IgG1-v34-LS	1 mg/kg	8.3

Table S4: Systematic search for covariates and correlations based on different doses and variants of eCD4Ig in the PK model that best recapitulates the observed data (model M9 in Table S1).

**Model**	**Comments**	**AIC ($\boldsymbol{\Delta AIC}$)**
Original Model M9 from Table S1 $\frac{dA_b}{dt} = -k_{bt}A_b + k_{tb}A_t - \frac{k_{bd}A_bA_d}{\left(1+\frac{A_b}{b_{50}f_b}\right)} - c_bA_b$ $\frac{dA_t}{dt} = k_{bt}A_b - k_{tb}A_t$	_This model_ assumes (i) ADA is produced in response to eCD4Ig in a linear fashion and this production rate has a negative feedback regulated by the amount of eCD4Ig. This term allows immune system to restrict from having a large number of ADA even when eCD4Ig keep increasing. There is	-203.4 (0)

$\frac{dA_d}{dt} = \lambda_d + \frac{r(t)A_b}{(d_{50}f_b + A_b)} - c_d A_d$<br>where,<br>$r(t) = 0$ is $t < \tau$ but $r(t) = r_d$ if $t \geq \tau$	also an **on-off switch** that controls the production of ADA in response to eCD4Ig.<br>(ii) ADA mediates clearance of eCD4Ig antibodies, and this clearance rate gets saturated as eCD4Ig in the blood increase.	
Model M9-v1: Model M9+<br>*(i)Correlation between* k_{bt} and c_b	Monolix suggested correlation	-212 (9)
Model M9-v2: Model M9+<br>*(i)Correlation between* k_{bd} and λ_d	Monolix suggested correlation	-185.2 (9)
Model M9-v3: Model M9+<br>*(i) Correlation between* k_{bt} and c_b<br>(ii) *Correlation between* k_{bd} and λ_d	Monolix suggested correlation	-216.7 (13.7)
Model M9-v4: Model M9-v3+<br>*(i) Covariate on* c_b *based on dose and variant*	Covariates explored on c_b (based on Figs S1 and S2)	-19.52 (198)
Model M9-v5: Model M9-v3+<br>*(i) Covariate on* c_b *based on dose*	Covariates explored on c_b (based on Figs S1 and S2)	-71.8 (145)
Model M9-v6: Model M9-v3+<br>*(i) Covariate on* c_b *based on variant*	Covariates explored on c_b (based on Figs S1 and S2)	-61.6 (155)
Model M9-v7: Model M9 +<br>*(i) Covariate on* c_b *based on dose and variant*	Covariates explored on c_b (based on Figs S1 and S2)	-58.1 (145)
Model M9-v8: Model M9 +<br>*(i) Covariate on* c_b *based on dose*	Covariates explored on c_b (based on Figs S1 and S2)	-137.2 (66)
Model M9-v9: Model M9 +<br>*(i) Covariate on* c_b *based on variant*	Covariates explored on c_b (based on Figs S1 and S2)	-61.8 (141)
Model M9-v10: Model M9 +<br>*(i) Covariate on* k_{bd} *based on variant*	Covariates explored on k_{bd} (based on Fig S2)	-85.7 (117)
Model M9-v11: Model M9 +<br>*(i) Covariate on* k_{bd} *and* τ *based on variant*	Covariates explored on k_{bd} and τ (based on Fig S2)	-47.5 (155)
Model M9-v12: Model M9 +<br>*(i) Covariate on* k_{tb} *based on dose and variant*	Covariates explored on k_{tb} (based on Figs S1 and S2)	-163.7 (39)
Model M9-v13: Model M9 +<br>*(i) Covariate on* k_{tb} *based on variant*	Covariates explored on k_{tb} (based on Fig S2)	-27.7 (175)
Model M9-v14: Model M9 +<br>*(i) Covariate on* k_{bt} *based on dose and variant*	Covariates explored on k_{bt} (based on Figs S1 and S2)	-156.5 (46)

Table S5: The population level fixed value and the standard deviation of the random effects of all parameters in the PK model that best recapitulates the data (as model M9 in Table S1). These

parameters are estimated by fitting eCD4Ig and anti-drug antibodies data from 21 uninfected rhesus macaques in Monolix R2019R2.

Parameter	Fixed value	Standard deviation of the random effects
k_{bt}	0.63	0.67
k_{tb}	0.50	0.34
c_b	0.37	0.60
k_{bd}	0.09	1.24
b_{50}	0.14	0.99
r_d	93.3	0.58
c_d	0.0495	Fixed from the ref. (*15*)
λ_d	4.4	1.61
τ	9.3	0.02
d_{50}	0.08	2.3
Correlation	(k_{bt}, c_b)	(k_{bd}, λ_d)
	0.65	-0.94

Table S6: The estimated parameter values individually for each infected animal in the model that was described in the main text (and as model M9 in Table S1). These parameters are estimated by fitting eCD4Ig and anti-drug antibodies data from 21 uninfected rhesus macaques and eCD4Ig data from 12 infected rhesus macaques in Monolix R2019R2. We also fixed $c_d = 0.0495$/day. Here, $t_{1/2}$ represents the terminal half-life in days. All animals with * received the first dose of eCD4-IgG1-v34-LS and the third dose of eCD4IgG2-v26-LS, and a second dose of eCD4Ig variant as provided in the Table.

ID	k_{bt}	k_{tb}	c_b	k_{bd}	b_{50}	r_d	λ_d	τ	d_{50}	Protein	Dose	$t_{1/2}$
r13015	0.5	0.4	0.5	0.1	0.2	125.2	3.4	9.3	0.06	eCD4IgG2-v26-LS	30 mg/kg	4.1
r13044	0.6	0.3	0.5	0.1	0.1	94.0	4.4	9.3	0.07	eCD4IgG1-v34-LS	30 mg/kg	5.3
r13070	0.5	0.2	0.5	0.1	0.1	82.7	4.9	9.3	0.08	eCD4IgG1-v34-LS	30 mg/kg	7.5
r13092	0.6	0.3	0.6	0.1	0.1	77.5	5.1	9.3	0.09	eCD4IgG2-v26-LS	30 mg/kg	6.0
r12060*	0.9	0.4	1.2	0.1	0.1	105.1	4.0	9.3	0.07	eCD4IgG2-v26-LS	30 mg/kg	3.1
r13040*	0.7	0.2	0.9	0.1	0.6	105.8	3.9	9.3	1.01	eCD4IgG2-v26-LS	30 mg/kg	7.4
r13066*	0.5	0.1	0.4	0.1	0.3	102.2	4.3	9.3	0.01	eCD4IgG1-v34-LS	30 mg/kg	15.1
r13085*	0.7	0.4	0.9	0.2	0.2	145.9	2.9	9.3	0.03	eCD4IgG2-v26-LS	30 mg/kg	3.9
r14017*	0.5	0.2	0.5	0.1	0.1	101.7	4.2	9.3	0.01	eCD4IgG1-v34-LS	30 mg/kg	10.3
r14045*	0.7	0.2	0.7	0.1	0.8	97.5	4.3	9.3	0.11	eCD4IgG1-v34-LS	30 mg/kg	6.6

r14048*	0.6	0.3	0.6	0.1	0.2	109.4	3.9	9.3	0.08	eCD4IgG1-v34-LS	30 mg/kg	5.2
r14084*	0.5	0.2	0.7	0.1	0.7	77.0	4.8	9.3	2.09	eCD4IgG2-v26-LS	30 mg/kg	6.0

Table S7: The population level fixed value and the standard deviation of the random effects of all parameters in the PK model that was described in the main text (and as model M9 in Table S1). These parameters are estimated by fitting eCD4Ig and anti-drug antibodies data from 21 uninfected rhesus macaques and 12 infected animals in Monolix R2019R2. * represents those parameters that were assumed to have the same population distribution obtained from fitting from 21 uninfected rhesus macaques because ADA data is not available for infected animals.

Parameter	Fixed value	Standard deviation of the random effects
k_{bt}	0.63 *	0.32
k_{tb}	0.50 * (U) & 0.25 (I)	0.54
c_b	0.37 * (U) & 0.68 (I)	0.42
k_{bd}	0.09 *	0.71
b_{50}	0.14 *	1.07
r_d	93.3 *	0.58 *
c_d	0.0495 *	Fixed from the ref. (*15*)
λ_d	4.4 *	1.61 *
τ	9.3 *	0.02 *
d_{50}	0.08 *	2.3 *
Correlation	(k_{bt}, c_b)	(k_{bd}, λ_d)
	0.71	-0.98
Covariate on k_{tb}	-0.686	
Covariate on c_b	0.604	

Table S8: The performance of models in reproducing the viral dynamics. The PK parameters were kept fixed from Table S5 for infected animals. Here, ΔAIC represents the difference between AIC of a model and the AIC of the best model.

Model	Structure	Parameters	AIC ($\boldsymbol{\Delta AIC}$)
MP1	<u>Model without eclipse phase</u><br>$\frac{dT}{dt} = \lambda_T - d_T T - \beta V T$<br>$\frac{dI}{dt} = a_L + \beta V T - d_I I - \delta_{IE} I E$<br>$\frac{dV}{dt} = pI - cV$<br>$\frac{dE}{dt} = \lambda_E + \frac{\Omega I E}{I + I_{50}} - d_E E$	• d_I=0.4/day (*47-49*)<br>• $d_M = d_T$	1008.6 (119.8)

MP2	Model with eclipse phase<br>$\frac{dT}{dt} = \lambda_T - d_T T - \beta VT$<br>$\frac{dM}{dt} = \beta VT - aM - d_M M$<br>$\frac{dI}{dt} = a_L + aM - d_I I - \delta_{IE} IE$<br>$\frac{dV}{dt} = pI - cV$<br>$\frac{dE}{dt} = \lambda_E + \frac{\Omega IE}{I+I_{50}} - d_E E$	• d_I=0.8/day (*41*)<br>• $d_M = d_T$	938.2 (49.4)
MP3	Model MP2	• d_I=0.8/day (*41*)<br>• $d_M = d_T$	948.8 (60)
MP4	Model MP2	• d_I=0.4/day (*47-49*)<br>• $d_M = d_T$<br>• δ_{IE} are different pre- and post-ART interruption (*21*)	908.4 (19.6)
MP5	Model MP2	• d_I=0.4/day (*47-49*)<br>• $d_M = d_T$<br>• β are different pre- and post-ART interruption (*21*)	898.7 (9.9)
MP6	Model MP2	• d_I=0.4/day (*47-49*)<br>• $d_M = d_T$<br>• β and δ_{IE} are different pre- and post-ART interruption (*21*)	888.8 (0)
MP7	Model MP2	• d_I=0.4/day (*47-49*)<br>• $d_M = d_T$<br>• β, p and δ_{IE} are different pre- and post-ART interruption (*21*)	907.6 (18.8)
MP8	Model MP2	• d_I=0.4/day (*47-49*)<br>• $d_M = 0.066\ (0.038)$ (*58*)<br>• β and δ_{IE} are different pre- and post-ART interruption (*21*)	945.6 (56.8)
MP9	Model MP2	• d_I=0.4/day (*47-49*)<br>• $d_M = d_T$<br>• β and δ_{IE} are different pre- and post-ART interruption (*21*)<br>• EC_{50} follows a different distribution based on the phase of infection	894.3 (5.5)
MP10	Model with eclipse phase and with short and long-lived infected cells (*41, 58*)<br>$\frac{dT}{dt} = \lambda_T - d_T T - \beta VT$<br>$\frac{dM}{dt} = \beta VT - aM - d_M M$<br>$\frac{dI_S}{dt} = f_L a_L + faM - d_I I_S - \delta_{IE} I_S E$	• d_I=0.4/day (*47-49*)<br>• $d_M = d_T$<br>• $f > 0.99$ (*41, 58*)<br>• β and δ_{IE} are different pre- and post-ART interruption (*21*)	977.7 (88.9)

	$\frac{dI_L}{dt} = (1-f_L)a_L + (1-f)aM - d_I I_L - \delta_{IE} I_L E$<br>$\frac{dV}{dt} = p(I_S + I_L) - cV$<br>$\frac{dE}{dt} = \lambda_E + \frac{\Omega(I_S+I_L)E}{(I_S+I_L)+I_{50}} - d_E E$	• EC_{50} follows a different distribution based on the phase of infection	
MP11	Model MP10	• d_I=0.8/day (*41*)<br>• $d_M = d_T$<br>• $f > 0.99$ (*41, 58*)<br>• β and δ_{IE} are different pre- and post-ART interruption (*21*)<br>• EC_{50} follows a different distribution based on the phase of infection	921.4<br>(32.6)

Table S9: Individual parameter values from the best fits of the viral dynamics model. We fixed $p = 50{,}000$ virions/day/infected-cell (*51*), a=1/day (*59*), $d_I = 0.4$/day (*47-49*), c =23/day (*50*), $\epsilon_{ART} = 0.99$ (*40*) and $d_E = 0.003$/day (*21*). We also estimated $d_T = 0.1$/day and $a_L = 10^{-2}$ cells/μL/day. The units for λ_T, β, δ_{IE}, λ_E, Ω, I_{50} and IC_{50} are cells/μL/day, μL/virions/day, μL/day/cells, cells/μL/day, μL/day/cells, cells/μL and $\mu g/mL$, respectively, whereas ϕ, β^*, δ_{IE}^* are unitless.

ID	λ_T (x10[3])	β (x10[-5])	δ_{IE}	λ_E (x10[-4])	Ω	I_{50}	IC_{50}	ϕ (x10[-7])	β^*	δ_{IE}^*
r13015	1.95	1.04	19.5	1.47	0.12	9.8	25.1	2.1	11.0	3.6
r13044	2.91	0.26	8.0	1.42	0.09	56.2	31.6	2.1	1.7	25.5
r13070	1.61	1.76	25.6	1.48	0.14	39.0	25.1	1.6	5.0	56.6
r13092	1.91	1.11	18.9	1.46	0.08	4.5	25.1	1.9	18.8	1.3
r12060	2.93	0.54	12.0	1.44	0.10	43.1	25.1	2.0	-	-
r13040	3.54	4.27	8.9	1.43	0.13	2.9	25.1	2.3	-	-
r13066	3.04	0.72	12.0	1.44	0.11	41.4	25.1	1.9	-	-
r13085	3.37	0.35	9.9	1.43	0.09	39.7	25.1	2.0	-	-
r14017	4.62	0.39	7.0	1.42	0.08	43.6	25.1	2.0	-	-
r14045	5.21	5.34	6.6	1.41	0.11	13.8	31.6	2.0	-	-
r14048	3.62	10.12	8.9	1.43	0.16	2.6	31.6	1.9	-	-
r14084	2.77	0.28	12.8	1.45	0.11	166.6	25.1	2.0	-	-
**Median**	2.99	0.9	11.0	1.44	0.11	39.4	25.1	2.0	8.0	14.6

Table S10: List of models explored to reproduce the dynamics of eCD4Ig in the blood (A_b) and anti-drug antibodies in the blood (A_d) in 8 uninfected rhesus macaques after a single administration of 2.5×10[13] AAV1-delivered rh-eCD4IgI39N (*7, 10*). As a starting point, we borrowed the best model from **Table S1** (model M9). As a revision, in this basic, A_b and A_t denote the amount of

eCD4Ig in blood and tissue (or some other compartment), respectively. Similarly, $\mathrm{A_d}$ represent the amount of anti-drug antibodies (ADA) in the blood. Among these models, the movement of eCD4Ig between the blood and the tissue compartment is modelled through rates $\mathrm{k_{bt}}$ and $\mathrm{k_{tb}}$ whereas eCD4Ig are cleared from the blood at rate $\mathrm{c_b}$. There is also a background production rate of ADA at rate λ_d and a clearance rate of $\mathrm{c_d}$. There is also an **on-off switch** denoted by $r(t)$ that controls the production of ADA in response to eCD4Ig in a linear fashion that gets saturated by the amount of eCD4Ig (d_{50}). Finally, ADA mediates clearance of eCD4Ig antibodies (k_{bd}), and this clearance rate gets saturated as eCD4Ig in the blood increase (b_{50}). The new components in the model (MA1) include, (i) the number of AAV vectors administered (V_{AAV}), (ii) the number of target muscle cells that are transduced with AAV vectors (I_{AAV}), and (iii) the transient immune response mounted potentially in the form of NK cells against muscle cells transduced with AAV vectors (E_{NK}). The target muscle cells (T_{AAV}) that are assumed to remain constant (due to a short half-life of AAV vectors) become transduced with AAV vector at rate β_{AAV}. The turnover rates of AAV vectors and target muscle cells are denoted by d_{AAV} and δ_{AAV}, respectively, whereas cells transduced by helper viruses such as adenoviruses likely die at an additional rate of δ_N as a result of AAV replication (*44*) and due to the immune responses such as NK cells elicited by AAV vectors (*26-29*). Moreover, we assume that AAV vector transduced muscle cells produce eCD4Ig at rate p_{AAV}. While there is a background production rate of immune response such as NK cells at rate λ_N and a clearance rate of c_N in the absence of AAV vectors, the presence of AAV facilitate the expansion of such responses at a rate ζ, which is assumed to get saturated at high numbers of AAV vector transduced muscle cells (controlled by the parameter n_{50}). We have initial conditions as, $V_{AAV}(0) = 2.5 \times 10^{13}$ AAV vectors, $I_{AAV}(0) = 0$ cells, $E_{NK}(0) = \frac{\lambda_N}{c_N}$ and $T_{AAV} = 3.7 \times 10^{12}$ cells (based on 3.7×10^{13} cells in a human adult body (*60*) and the weight difference of ~10 fold between humans and rhesus macaques). The number of target muscle cells are assumed constant as AAV vectors deplete quickly with a short half-life of $d_{AAV} = 24 \times \frac{0.69}{2}$ /day (*54*). We further assume $\lambda_N = 0.0001$ cells/day (reflecting the presence of NK cells at a very low level at $t = 0$), and $c_N = \frac{0.69}{14}$ /day (*55, 56*). Moreover, we fixed δ_{AAV}=0.0003/day under the assumption that muscle cells live for a long time (~10 years) (*57*). We also assume the weight of all animals as $W = 8$ kg, which is then used to calculate their volume of the blood as $60 \times W$ mL. It is further assumed that NK cells do not influence ADA dynamics and ADA does not influences NK dynamics. Specific model related components and assumptions are described in comments section in this Table. The best performing model is highlighted in red.

**Model**	**Comments**	**AIC** **ΔAIC**
<u>*Model MA1*</u> $\frac{dV_{AAV}}{dt} = -\beta_{AAV}V_{AAV}T_{AAV} - d_{AAV}V_{AAV}$ $\frac{dI_{AAV}}{dt} = \beta_{AAV}V_{AAV}T_{AAV} - \delta_{AAV}I_{AAV} - \delta_N E_{NK} I_{AAV}$ $\frac{dA_b}{dt} = p_{AAV}I_{AAV} - k_{bt}A_b + k_{tb}A_t - \frac{k_{bd}A_bA_d}{\left(1+\frac{A_b}{b_{50}f_b}\right)} - c_bA_b$	<u>*Best performing model*</u>	-115.3 (0)

<table>
<tr><td>
$$\frac{dA_t}{dt} = k_{bt}A_b - k_{tb}A_t$$
$$\frac{dA_d}{dt} = \lambda_d + \frac{r(t)A_b}{(d_{50}f_b + A_b)} - \frac{s(t)A_b}{(e_{50}f_b + A_b)} - c_dA_d$$
$$\frac{dE_{NK}}{dt} = \lambda_N + \frac{\zeta E_{NK}I_{AAV}}{(n_{50} + I_{AAV})} - c_NE_{NK}$$
where,
$r(t) = 0$ is $t < \tau$ but $r(t) = r_d$ if $t \geq \tau$
$s(t) = 0$ is $t < \tau$ but $s(t) = s_d$ if $t \geq \tau$
<u>Number of unknown parameters</u>: 15
</td><td></td><td></td></tr>
<tr><td>
<u>Model MA2</u>
$$\frac{dV_{AAV}}{dt} = -\beta_{AAV}V_{AAV}T_{AAV} - d_{AAV}V_{AAV}$$
$$\frac{dI_{AAV}}{dt} = \beta_{AAV}V_{AAV}T_{AAV} - \delta_{AAV}I_{AAV} - \delta_NE_{NK}I_{AAV}$$
$$\frac{dA_b}{dt} = p_{AAV}I_{AAV} - k_{bt}A_b + k_{tb}A_t - \frac{k_{bd}A_bA_d}{\left(1 + \frac{A_b}{b_{50}f_b}\right)} - c_bA_b$$
$$\frac{dA_t}{dt} = k_{bt}A_b - k_{tb}A_t$$
$$\frac{dA_d}{dt} = \lambda_d + \frac{r(t)A_b}{(d_{50}f_b + A_b)} - c_dA_d$$
$$\frac{dE_{NK}}{dt} = \lambda_N + \frac{\zeta E_{NK}I_{AAV}}{(n_{50} + I_{AAV})} - c_NE_{NK}$$
where,
$r(t) = 0$ is $t < \tau$ but $r(t) = r_d$ if $t \geq \tau$
<u>Number of unknown parameters</u>: 13
</td><td><u>Model MA2</u> assumes that there is no saturation of ADA in model MA1 (i.e., $s_d = 0$).</td><td>102.6 (217.9)</td></tr>
<tr><td>
<u>Model MA3</u>
$$\frac{dV_{AAV}}{dt} = -\beta_{AAV}V_{AAV}T_{AAV} - d_{AAV}V_{AAV}$$
$$\frac{dI_{AAV}}{dt} = \beta_{AAV}V_{AAV}T_{AAV} - \delta_{AAV}I_{AAV} - \delta_NE_{NK}I_{AAV}$$
$$\frac{dA_b}{dt} = p_{AAV}I_{AAV} - \frac{k_{bd}A_bA_d}{\left(1 + \frac{A_b}{b_{50}f_b}\right)} - c_bA_b$$
$$\frac{dA_t}{dt} = k_{bt}A_b - k_{tb}A_t$$
$$\frac{dA_d}{dt} = \lambda_d + \frac{r(t)A_b}{(d_{50}f_b + A_b)} - \frac{s(t)A_b}{(e_{50}f_b + A_b)} - c_dA_d$$
$$\frac{dE_{NK}}{dt} = \lambda_N + \frac{\zeta E_{NK}I_{AAV}}{(n_{50} + I_{AAV})} - c_NE_{NK}$$
where,
$r(t) = 0$ is $t < \tau$ but $r(t) = r_d$ if $t \geq \tau$
$s(t) = 0$ is $t < \tau$ but $s(t) = s_d$ if $t \geq \tau$
</td><td><u>Model MA3</u> assumes that there is no intercompartmental exchange of eCD4Ig in model MA1 (i.e., $k_{bt} = 0$ and $k_{tb} = 0$).</td><td>16.3 (131.6)</td></tr>
</table>

Number of unknown parameters: 13		
Model MA4<br>$$\frac{dV_{AAV}}{dt} = -\beta_{AAV}V_{AAV}T_{AAV} - d_{AAV}V_{AAV}$$<br>$$\frac{dI_{AAV}}{dt} = \beta_{AAV}V_{AAV}T_{AAV} - \delta_{AAV}I_{AAV}$$<br>$$\frac{dA_b}{dt} = p_{AAV}I_{AAV} - k_{bt}A_b + k_{tb}A_t - \frac{k_{bd}A_bA_d}{\left(1+\frac{A_b}{b_{50}f_b}\right)} - c_bA_b$$<br>$$\frac{dA_t}{dt} = k_{bt}A_b - k_{tb}A_t$$<br>$$\frac{dA_d}{dt} = \lambda_d + \frac{r(t)A_b}{(d_{50}f_b + A_b)} - \frac{s(t)A_b}{(e_{50}f_b + A_b)} - c_dA_d$$<br>where,<br>$r(t) = 0$ is $t < \tau$ but $r(t) = r_d$ if $t \geq \tau$<br>$s(t) = 0$ is $t < \tau$ but $s(t) = s_d$ if $t \geq \tau$<br>*Number of unknown parameters*: 12	*Model MA4* assumes that there are no E_{NK} cells in model MA1 (i.e., $E_{NK} =$ 0 and δ_N=0).	110.6<br>(225.9)
Model MA5<br>$$\frac{dV_{AAV}}{dt} = -\beta_{AAV}V_{AAV}T_{AAV} - d_{AAV}V_{AAV}$$<br>$$\frac{dI_{AAV}}{dt} = \beta_{AAV}V_{AAV}T_{AAV} - \delta_{AAV}I_{AAV} - \delta_NI_{AAV}$$<br>$$\frac{dA_b}{dt} = p_{AAV}I_{AAV} - k_{bt}A_b + k_{tb}A_t - \frac{k_{bd}A_bA_d}{\left(1+\frac{A_b}{b_{50}f_b}\right)} - c_bA_b$$<br>$$\frac{dA_t}{dt} = k_{bt}A_b - k_{tb}A_t$$<br>$$\frac{dA_d}{dt} = \lambda_d + \frac{r(t)A_b}{(d_{50}f_b + A_b)} - \frac{s(t)A_b}{(e_{50}f_b + A_b)} - c_dA_d$$<br>where,<br>$r(t) = 0$ is $t < \tau$ but $r(t) = r_d$ if $t \geq \tau$<br>$s(t) = 0$ is $t < \tau$ but $s(t) = s_d$ if $t \geq \tau$<br>*Number of unknown parameters*: 12	*Model MA5* assumes that there are no E_{NK} cells in model MA1 (i.e., $E_{NK} =$ 0 and δ_N≠0).	39.3<br>(154.6)
Model MA6<br>$$\frac{dV_{AAV}}{dt} = -\beta_{AAV}V_{AAV}T_{AAV} - d_{AAV}V_{AAV}$$<br>$$\frac{dM_{AAV}}{dt} = \beta_{AAV}V_{AAV}T_{AAV} - a_{AAV}M_{AAV}$$<br>$$\frac{dI_{AAV}}{dt} = a_{AAV}M_{AAV} - \delta_{AAV}I_{AAV} - \delta_NE_{NK}I_{AAV}$$<br>$$\frac{dA_b}{dt} = p_{AAV}I_{AAV} - k_{bt}A_b + k_{tb}A_t - \frac{k_{bd}A_bA_d}{\left(1+\frac{A_b}{b_{50}f_b}\right)} - c_bA_b$$	*Model MA6* assumes that before infected muscles cells start producing eCD4Ig, they go through an addition eclipse phase (M_{AAV}) in model MA1 and these non-productive infected muscle cells become productive at rate a_{AAV}.	-41.8<br>(73.5)

$\frac{dA_t}{dt} = k_{bt}A_b - k_{tb}A_t$<br>$\frac{dA_d}{dt} = \lambda_d + \frac{r(t)A_b}{(d_{50}f_b + A_b)} - \frac{s(t)A_b}{(e_{50}f_b + A_b)} - c_dA_d$<br>$\frac{dE_{NK}}{dt} = \lambda_N + \frac{\zeta E_{NK}I_{AAV}}{(n_{50} + I_{AAV})} - c_NE_{NK}$<br>where,<br>$r(t) = 0$ is $t < \tau$ but $r(t) = r_d$ if $t \geq \tau$<br>$s(t) = 0$ is $t < \tau$ but $s(t) = s_d$ if $t \geq \tau$<br>_Number of unknown parameters_: 16		

Table S11: The estimated parameter values individually for each infected animal in the model that was described in the main text (and as model MA1 in Table S10). These parameters are estimated by fitting eCD4Ig and anti-drug antibodies data from 8 uninfected rhesus macaques that were administered 2.5×10^{13} AAV vectors at time $t = 0$. The fitting was performed in Monolix R2019R2. We also fixed $c_d = 0.0495$/day and $c_b = 0.37$/day. We also fixed $d_{AAV} = 24 \times \frac{0.69}{2}$/day, $\lambda_N = 0.0001$ cells/day, $c_N = \frac{0.69}{14}$/day and δ_{AAV}=0.0003/day.

ID	k_{bt}	k_{tb}	k_{bd}	b_{50}	r_d	λ_d	τ	d_{50}	β_{AAV} ($\times10^{-24}$)	δ_N	p_{AAV} ($\times10^{3}$)	s_d	e_{50} ($\times10^{-6}$)	ζ	n_{50} ($\times10^{2}$)
2448	164.4	0.37	0.07	1.6	418.8	5.5	17.4	11.2	0.30	0.07	6.2	108.6	1.5	0.47	1.5
12080	159.4	0.37	0.07	1.5	343.3	3.5	17.2	36.2	0.28	0.33	13.7	117.5	1.6	0.55	1.4
13023	154.3	0.37	0.07	1.6	342.6	4.9	17.3	16.6	0.29	0.48	11.2	107.4	1.5	0.50	1.5
13058	133.6	0.37	0.07	1.7	380.9	8.4	17.3	39.0	0.29	0.60	14.3	117.8	1.5	0.49	1.5
180-10	153.1	0.37	0.07	1.6	179.5	11.2	17.3	15.7	0.30	0.24	14.6	119.4	0.4	0.43	1.6
181-10	147.0	0.37	0.07	1.6	236.9	11.9	17.4	61.5	0.29	0.18	18.5	125.4	1.9	0.48	1.5
265-10	166.9	0.37	0.07	1.5	279.7	11.2	17.4	30.9	0.30	0.13	14.3	113.6	1.1	0.44	1.6
431-10	158.3	0.37	0.07	1.4	248.2	9.8	17.3	58.3	0.30	0.12	21.2	123.7	1.6	0.43	1.6
